# Advances in Silicone Implants Characterization: A Comprehensive Overview of Chemical, Physical and Biological Methods for Biocompatibility Assessment

**DOI:** 10.3390/bioengineering12121307

**Published:** 2025-11-28

**Authors:** Kevin Dzobo, Nonhlanhla Khumalo, Vanessa Zamora Mora, Audry Zoncsich, Roberto de Mezerville, Ardeshir Bayat

**Affiliations:** 1 Medical Research Council-SA Wound Healing Unit, Hair and Skin Research Laboratory, Division of Dermatology, Department of Medicine, Groote Schuur Hospital, Faculty of Health Sciences, University of Cape Town, Anzio Road, Observatory, Cape Town 7925, South Africa; kevin.dzobo@uct.ac.za (K.D.); n.khumalo@uct.ac.za (N.K.); 2 Establishment Labs Holdings, Alajuela 20113, Costa Rica; vzamora@establishmentlabs.com (V.Z.M.); azoncsich@establishmentlabs.com (A.Z.); rdemezerville@establishmentlabs.com (R.d.M.)

**Keywords:** silicone implants, biocompatibility, characterization, analytical techniques, in vitro assays, surface modifications, regulatory standards, emerging technologies, clinical outcomes

## Abstract

Silicone implants are widely used in medical applications, particularly for breast augmentation and reconstruction. However, ongoing concerns regarding their long-term safety and biocompatibility necessitate comprehensive characterization. This review critically evaluates the chemical, physical, and biological testing approaches currently used to assess silicone implants, and specifically silicone breast implants, biocompatibility, and highlights the limitations of existing ISO 10993-based protocols, which often apply a one-size-fits-all model. We propose an application-specific framework to improve the relevance and precision of biocompatibility assessments. Chemical analyses, including Fourier transform infrared (FTIR) spectroscopy, Raman spectroscopy, and nuclear magnetic resonance (NMR) spectroscopy, provide essential information on polymer structure, integrity, and composition, thereby supporting quality control and market surveillance. Physical characterization methods, such as scanning electron microscopy (SEM), atomic force microscopy (AFM), and contact angle measurements, assess the surface morphology, hydrophobicity, and potential defects that may influence the host response. Mechanical testing, which evaluates properties such as tensile strength and fatigue resistance, simulates in vivo stress conditions to predict the long-term durability. Biological evaluations guided by ISO 10993 use in vitro and in vivo models to assess cytotoxicity, adhesion, inflammation, and tissue integration. However, these are often not tailored to the implant type, surface features, or duration of exposure. Emerging tools, such as organ-on-a-chip platforms and machine learning models, offer new possibilities for predictive and context-specific evaluation. We advocate a standardized, modular strategy that integrates chemical, physical, and biological testing with clinical data to bridge preclinical assessments and real-world outcomes, with a specific focus on silicone breast implants. The aim of this approach is to improve patient safety, regulatory clarity, and device innovation across the global landscape of silicone implant development.

## 1. Introduction

Silicone implants have become the mainstay in both cosmetic and reconstructive surgeries, particularly breast augmentation and reconstruction procedures [1]. Silicone implants are categorized primarily by the average shell surface roughness and filler gel cohesivity across generational advancements. The shell surface types include smooth (minimal roughness, Ra < 10 μm), micro-textured (intermediate roughness, Ra: 10–100 μm), and macrotextured (aggressive roughness, Ra > 50 μm) (ISO 14607:2018) [2]. Filler materials have evolved from low-cohesivity viscous gels in first- and second-generation implants (which are prone to leakage) to highly cohesive, form-stable gels found in fifth- and sixth-generation variants [3,4]. The differences between silicone implants profoundly influence their biocompatibility. Smoother surfaces promote denser collagen alignment, elevating the risk of capsular contracture (CC) via heightened inflammation, while textured topographies disrupt fibrosis, lowering CC incidence by 5–10% but increasing bacterial adhesion and rare complications, such as breast implant-associated anaplastic large-cell lymphoma (BIA-ALCL), mostly in macrotextured designs [5]. Enhanced gel cohesivity minimizes leakage-induced chronic inflammation and improves long-term tissue integration. Modifications, such as drug coatings, suppress macrophage activation and cytokine release (e.g., interleukin 4 (IL-4) and transforming growth factor-β (TGF-β)), further mitigating fibrosis [6]. All silicone implants invariably elicit a foreign body response (FBR), initiated by protein adsorption, acute/chronic inflammation, foreign body giant cell formation, and inevitable fibrous encapsulation as a host defense mechanism [7]. Although surface and filler optimizations attenuate FBR severity and CC (incidence approximately 10.6%), complete elimination remains unattainable, underscoring the need for ongoing biomimetic innovations.

The widespread use of silicone implants is attributed to their perceived inertness, flexibility, low toxicity, and compatibility with human tissues. In 2023 alone, the American Society of Plastic Surgeons (ASPS) reported over 300,000 breast augmentation procedures in the United States, with the majority utilizing silicone-based implants [8,9]. Beyond cosmetics, silicone’s role in ophthalmology and orthopedics underscores its versatility [10]. For example, in ophthalmology, intraocular lenses made of silicone are used to replace the natural lens, most commonly during cataract surgery, to restore clear vision. Silicone spacers have been used in orthopedic surgeries of the wrists and fingers, primarily in procedures to treat arthritis and joint damage. Silicone implant biocompatibility is key to its use, as it influences both patient safety and the success of surgical interventions [11,12]. Early silicone implants were relatively simple, but recently introduced implants have shown great complexity, including multilayered shells, anatomical shapes, a highly cohesive gel, and a visual barrier layer to aid in detecting leakage. These new silicone implants have raised concerns regarding potential adverse systemic responses and complications in patients over their intended lifespan [13]. Key historical events and biocompatibility controversies have led to both regulatory and methodological advancements required for precise biocompatibility characterization to meet stringent standards and address global regulatory requirements (Table 1) [14,15,16].

The controversies regarding the biocompatibility of silicone implants necessitate more comprehensive evaluations of the effects of silicone implants throughout the body, and advanced methods and techniques are under development for the accurate assessment of biocompatibility [14,15,16]. Controversies during the early stages of silicone implant evolution, including the Dow Corning litigation in the 1990s, in which silicone breast implants were linked to various health issues, underscore the need for better and more transparent biocompatibility evaluation techniques and methods (Table 1) [17,18]. In some cases, the FDA issues only cautionary statements to the public and healthcare providers regarding the association between silicone implants and adverse health conditions. An example is breast implant-associated squamous cell carcinoma. The first reported case of breast implant-associated squamous cell carcinoma (BIA-SCC) was documented in 1992, involving a silicone gel-filled implant placed for augmentation, with symptoms appearing 15 years after implantation [19]. Subsequent case reports have shown associations with both silicone and saline implants (the latter of which have silicone outer shells), with a mean latency period of approximately 21 years from implantation to symptom onset or diagnosis [19]. In response, the FDA issued an initial safety communication on 8 September 2022, alerting the public and healthcare providers to reports of SCC and other cancers (distinct from breast implant-associated anaplastic large cell lymphoma) developing in the scar capsule around breast implants (FDA Communication, updated March 2023) [20]. In this case, no broader policy changes, such as recalls or bans, were implemented.

Currently, the characterization of silicone implants involves a multifaceted approach encompassing chemical, physical, and biological assessments to ensure their safety, efficacy, and durability [21]. In most cases, the methods for characterization and regulatory standards are limited. A comprehensive evaluation is critical for predicting long-term performance and integrating clinical insights with preclinical data.

**Figure 1:**
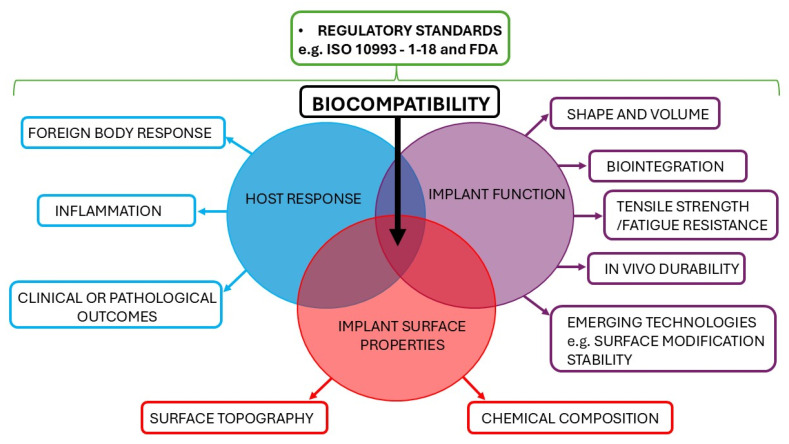
Silicone Implant Biocompatibility: A schematic illustrating the interplay between implant surface properties (red), host tissue/cell responses (blue), and implant function (purple), with biocompatibility at the center. Implant surface properties include texture, wettability, and chemical composition (assessed via chemical and physical methods); host responses, including inflammation, fibrosis, and foreign body response (assessed via biological methods); and function, including biointegration, tensile strength/fatigue resistance, and in vivo durability (assessed via physical and biological methods). Regulatory standards (e.g., ISO 10993) influence all aspects, whereas emerging technologies (e.g., organ-on-a-chip) offer future directions for integrated assessment.

Biocompatibility is a multifaceted concept that involves various interactions between an implant material and host tissue (Figure 1) [22,23]. It is defined by ISO 10993-1:2018 [24] as the “ability of a medical device or material to perform with an appropriate host response in a specific application” and is critical for patient safety and implant success [23]. It encompasses molecular-level toxicity and inflammation-to-tissue integration and requires a tailored approach for different implant types and applications. The interplay between surface properties, host tissue responses, and implant function results in what is termed biocompatibility (depicted as the intersection of host response, implant surface properties, and function). Surface properties, such as texture, wettability, and chemical composition, influence host responses, including fibrosis and inflammation. Overall, regulatory standards, including FDA standards and ISO 10993, govern all aspects of biocompatibility testing.

### 1.1. Silicone Implant Biocompatibility Assessment

Biocompatibility assessment involves both material characterization and biological testing, with the aim of understanding the properties of implants and predicting their biological performance [22,23]. For silicone implants, this involves ensuring that they do not provoke significant inflammatory responses, degrade into harmful by-products, ‘bleed’ excessively, or fail to integrate well with the surrounding tissue [6]. The biocompatibility of silicone implants is a critical factor that influences the extent of foreign body response and potential complications, such as breast implant-associated anaplastic large cell lymphoma (BIA-ALCL), and capsular contracture [25]. Regulatory bodies, including the European Medicines Agency (EMA) and the U.S. Food and Drug Administration (FDA), notified bodies such as TUV SUD (representing the European Union), and the International Organization for Standardization (ISO) have established frameworks, notably ISO 10993, to guide this process [16,26,27]. These standards encompass various tests designed to assess endpoints, such as cytotoxicity, genotoxicity, systemic toxicity, and implantation effects [27,28]. However, as silicone implants evolve with features such as micro-textured surfaces, nanoscale coatings (including polymer-based layers such as polyethylene glycol (PEG) and hyaluronic acid (HA), which create hydrophilic, antifouling surfaces via grafting or layer-by-layer (LBL) deposition), and modified chemistries (involving surface activation techniques such as plasma oxidation, UV treatment, or carbon-ion implantation to introduce reactive groups for covalent attachment of drugs or polymers), these generic protocols increasingly reveal their limitations. The primary gap addressed in this review is the lack of a systematic, application-specific framework for selecting the most relevant biocompatibility assessments for the diverse range of silicone implants available.

Briefly, the chemical characterization of silicone implants is essential to ensure their quality and safety, focusing on understanding their surface chemistry, which directly interacts with biological tissues and bodily fluids, the stability of silicone polymers, and the presence of leachable compounds or degradation products [29,30]. Spectroscopic techniques including Fourier Transformed Infrared spectroscopy (FTIR), Raman spectroscopy, and nuclear magnetic resonance (NMR) spectroscopy, are utilized to characterize the polymer surface chemistry, ensuring that no unintended functional groups emerge during manufacturing or degradation, and to distinguish between various brands and different silicone-based breast implants based on their chemical compositions [31,32,33].

In brief, the physical characterization of silicone implants involves assessing their mechanical properties and surface characteristics [16,27,28,34]. Mechanical testing assesses the elasticity, tensile strength, and fatigue resistance of silicone, ensuring that the implants can withstand the physiological stresses encountered [35]. The assessment of the biocompatibility of silicone implants extends beyond chemical and physical characterization to include in vitro and in vivo biological studies [36]. These evaluations are integral to biocompatibility assessment and typically involve cell culture experiments to assess cytotoxicity and cell adhesion, as well as animal studies to examine tissue responses and potential systemic effects [37]. In vitro tests, such as the MTT assay (ISO 10993-5), measure cytotoxicity by exposing cell lines (e.g., fibroblasts or macrophages) to the silicone implant surface or extracts and by gauging cell viability. In vivo implantation studies, often in models and human trials, can evaluate long-term outcomes, and histomorphometric analysis of explanted tissues can reveal factors such as fibrous capsule thickness and inflammatory cell infiltration [16,38]. These in vivo studies offer a more holistic view of how implants might behave over time by observing phenomena such as foreign body response, integration, and rejection.

### 1.2. Aim of the Review Manuscript

Despite this robust set of techniques, biocompatibility assessment faces a persistent challenge: the lack of a systematic approach for selecting the most relevant tests for a specific silicone implant. Although ISO 10993 provides a general roadmap, critics argue that it is a one-size-fits-all strategy that does not adequately account for the diversity of silicone types and applications [39,40]. A textured breast implant demands different scrutiny, such as macrophage assays or chronic implantation studies, compared to a smooth ocular implant, where epithelial viability is paramount [41]. Similarly, short-term subcutaneous fillers require distinct evaluations compared with long-term joint implants. This non-specific approach risks both over-testing, wasting time and resources, and under-testing, missing critical safety signals. Studies using 3D skin models have demonstrated the ability of silicone to upregulate pro-inflammatory cytokines, such as IL-6, without killing cells, suggesting that subtle chronic risks are not always captured by standard cytotoxicity tests [42]. Histomorphometric analyses in rats reveal that surface texture drives capsule formation, yet these findings rarely inform test selection beyond generic implantation studies [43]. Clinical reports, such as the FDA 2022 update linking mostly textured implants to ALCL, have revealed gaps between preclinical data and real-world outcomes and insufficient personalization for patient variability, such as genetic predisposition to inflammation [44]. Long-term data remain particularly scarce, with most tests focusing on acute or subchronic effects, leaving the decades-long fate of implants understudied. Both manufacturers and scientists often use available methods for implant/medical device characterization, including the analysis of chemical leachables, physical roughness, and biological inflammation, often with no clear application-specific characterization strategy, a problem compounded by the fact that silicone innovations outpace testing paradigms.

Advances in breast implant surface technologies include the modification of implant surface topography and chemistry to enhance biocompatibility and integration [6,25,45]. These modifications aim to optimize properties such as surface roughness, wettability, and stiffness, which directly affect cell-surface interactions, protein adsorption, and cell adhesion [46], respectively. With advances in silicone implant surface modification, there is a need for novel and improved characterization methods to ensure that ‘new’ silicone implant surfaces have the necessary biocompatibility for biointegration. The integration of these chemical, physical, and biological assessments into a coherent strategy for evaluating biocompatibility is crucial. Such an approach is needed to avoid overburdening implant development with redundant tests or overlooking hazards that may emerge years later. We propose a new framework based on adaptive tiered protocols, starting with chemical and physical assessments, including the use of artificial intelligence-driven predictive modeling to prioritize tests, followed by biological validation via in vitro assays and human-relevant models, such as organ-on-a-chip. Our proposed framework aims to empower scientists, regulators, and manufacturers to make informed choices that enhance safety and innovation. Clinical reports, such as the FDA 2022 update linking mostly textured implants to ALCL, highlight the gaps between preclinical data and real-world outcomes [44]. This manuscript highlights the gap in silicone implant biocompatibility characterization and the need for a structured testing strategy designed for different implants rather than relying on the current potentially disjointed application of ‘isolated’ tests. This review proposes a standardized application-specific framework for optimizing biocompatibility assessments by incorporating global regulatory perspectives and clinical correlations.

## 2. Methodology

The articles used in the synthesis of this review were obtained through an electronic search of the scientific literature, including Scopus, Web of Science, and PubMed. The search included keywords such as silicone implants, biocompatibility, characterization, analytical techniques, in vitro assays, in vivo models, ex vivo models, surface modifications, and emerging technologies. Authors screened the identified articles to determine whether they met the inclusion criteria. Duplicate and non-English manuscripts were excluded. Additional searches included clinical trial databases and regulatory guidelines from regions beyond the US and Europe to ensure global relevance.

## 3. Biocompatibility: Regulation and Standards

Evaluating biocompatibility is an important step in the regulatory framework governing medical devices to ensure their safety and suitability for human use [47]. Various regulatory authorities are responsible for formulating the steps and guidelines for biocompatibility testing [27,48], with a primary focus on evaluating the potential biological risks associated with medical devices and their constituent materials. These include the FDA (US), ISO (global), Notified Bodies representing European regulations (e.g., TÜV SÜD), and regional bodies such as China’s NMPA and Brazil’s Agência Nacional de Vigilância Sanitária (ANVISA).

The ISO, FDA, and European regulatory systems (often certified by organizations such as TÜV SÜD) are key entities that contribute significantly to the establishment of standards for biocompatibility testing [27,49]. ISO is well known for developing global standards for a diverse array of products and services, including silicone implants [38,50]. The ISO developed the 10993 series, which is commonly used as a standard for evaluating implant biocompatibility, and provides a set of tests designed to identify the potential biological risks associated with such devices [51,52]. Many other regulatory bodies worldwide have recognized and implemented ISO 10993 [52].

The FDA is responsible for regulating the development and testing of medical devices in the USA, which is crucial for securing pre-market approval of medical products [53]. The FDA requires manufacturers and scientists to conduct biocompatibility tests to evaluate the potential adverse effects of a device on the human body [54], and these requirements often follow the ISO 10993 standard [52]. In Europe, medical device regulation (MDR) conformity assessment often involves notified bodies, such as TÜV SÜD, which offers certification and testing services specifically for medical devices [55]. Manufacturers submit their products for assessment to ensure adherence to European Union (EU) regulations [16]. Certification, such as CE marking facilitated by a Notified Body, indicates that a medical device has been rigorously assessed and complies with the safety and efficacy standards mandated by the European Union [56].

The “ISO 10993,” series is divided into multiple parts, each addressing different standards that focus on the biological evaluation of these devices and materials, primarily considering their potential biological effects on the health of patients or users [27]. ISO 10993 provides a standardized approach for the biocompatibility testing of medical equipment and materials [38,47,49,51]. Part 1 of the document is the ‘planning’ section and offers guidelines for selecting appropriate tests based on a risk management process. In 2009, the ISO 10993-1 standard was revised to emphasize the use of chemical component testing (ISO-10993-18: Chemical characterization) and biological in vitro models, especially when these tests provide data similar to those obtained from in vivo models. This approach involves analyzing existing data before deciding whether further biocompatibility testing, such as animal studies, is necessary [38,47,49,51]. According to the available guidelines (both FDA guidelines and ISO 10993 standards), medical devices, including silicone implants, are classified based on three primary criteria: (1) nature of body contact (e.g., surface, external communicating, implant); (2) extent of contact between the material or device and the patient (limited, <24 h; prolonged, 24 h to 30 days; permanent, >30 days); and (3) the type of tissue contact (blood, tissues, or skin) [27]. Both quantitative and qualitative tests are recommended as part of biocompatibility testing, with emphasis on criteria such as possible immune reactions and duration of contact.

A key limitation of the FDA- and ISO-developed frameworks for conducting biocompatibility assessments is often perceived as the lack of mandated tests for different types of silicone implants [27,38,50,51]. Owing to the diversity in device categories, distinct devices, technologies, or materials require tailored testing, and varied biological endpoints may necessitate specific evaluations. Although the framework provides a matrix of potential tests, the selection requires justification based on the specific device and its intended use. However, guidance regarding tailoring is limited. Beyond the standard biological endpoints, the FDA and ISO emphasize the importance of assessing toxicity to developmental and reproductive organs, especially if the material has a known history of toxicity or if the device undergoes degradation in the body [27,49]. Similarly to the FDA and ISO frameworks, Notified Bodies, such as TÜV SÜD, assess the biocompatibility of devices and implants following the guidelines of ISO 10993 as part of the EU regulatory requirements [49,56]. TÜV SÜD operations typically comply with standards such as ISO 17025 [57] and Good Laboratory Practice (GLP), which are quality system standards that set the minimum criteria for conducting and reporting safety investigations [49,56].

Various regulatory authorities assert that these evaluations ensure that devices or materials that meet the requisite biocompatibility standards are deemed acceptable for their intended use in the human body. This assurance enables manufacturers to market their products with confidence, knowing that they have been subjected to a comprehensive biological risk assessment.

## 4. Chemical Methods for Characterization of Silicone Implants

**Figure 2:**
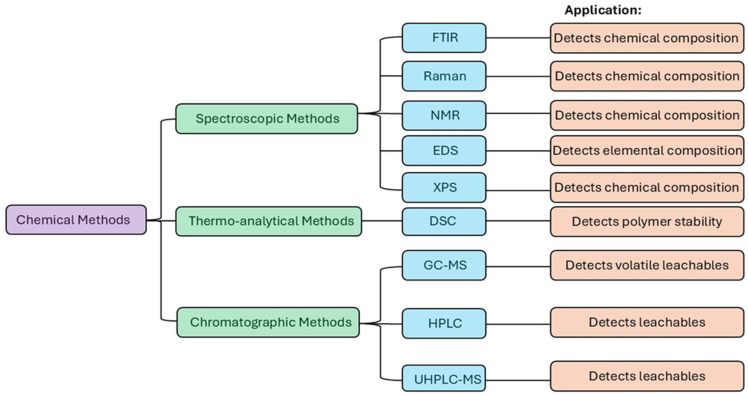
Classification of chemical characterization methods used to evaluate silicone implants. Spectroscopic methods, including FTIR, Raman spectroscopy, NMR spectroscopy, EDS, and XPS, provide insights into the molecular structure, functional groups, and elemental composition of surfaces. Chromatographic methods, including GC-MS, HPLC, and UHPLC-MS, can detect volatile, semi-volatile, and non-volatile leachables, extractables, and degradation products. Thermoanalytical methods, such as DSC, are used to assess polymer stability and phase transitions. Applications include FTIR for vinyl signals and GC-MS for low molecular weight silicones. Fourier transform infrared—FTIR spectroscopy; Raman spectroscopy—Raman; Nuclear magnetic resonance—NMR spectroscopy; Energy dispersive X-ray spectroscopy—EDS, X-ray photoelectron spectroscopy—XPS; Differential Scanning Calorimetry—DSC; Gas chromatography-Mass spectrometry—GC-MS; High performance liquid chromatography—HPLC; Ultra high-performance liquid chromatography-Mass spectrometry—UHPLC-MS.

Chemical characterization is essential for identifying the composition of silicone implants and detecting substances that may be toxic or provoke immune responses [58,59,60]. These methods are important to ensure the absence of leachables, degradation products, and impurities that could affect biocompatibility [61,62,63]. Chemical methods can detect specific features, such as surplus vinyl signals in the gel, cyclosiloxane impurities, and barrier layers in the implant envelope [33]. Techniques such as gas chromatography-mass spectrometry (GC-MS) and high-performance liquid chromatography (HPLC) (often coupled with MS (HPLC-MS)) are used to detect and quantify leachable and extractable substances that may raise toxicity concerns [34,64]. The recommended testing standards within the ISO 10993 series are parts 18 and 17 (establishment of allowable limits for leachable substances). Key techniques and methods include FTIR spectroscopy, Raman spectroscopy, NMR spectroscopy, energy dispersive X-ray spectroscopy (EDS), X-ray photoelectron spectroscopy (XPS), and GC-MS (Figure 2). These methods provide both quantitative and qualitative information about silicone implant composition, as described below. By linking the chemical composition, chemical stability, and surface properties of implants to biological outcomes, such as cell proliferation, adhesion, and inflammation, chemical techniques have advanced our understanding and development of biocompatible implants for both in vitro and in vivo applications (Table 2). Differential scanning calorimetry (DSC) is a thermo-analytical technique used to evaluate the stability and phase transition profiles of polymers used in implants.

### 4.1. Fourier Transform Infrared (FTIR) Spectroscopy

**Figure 3:**
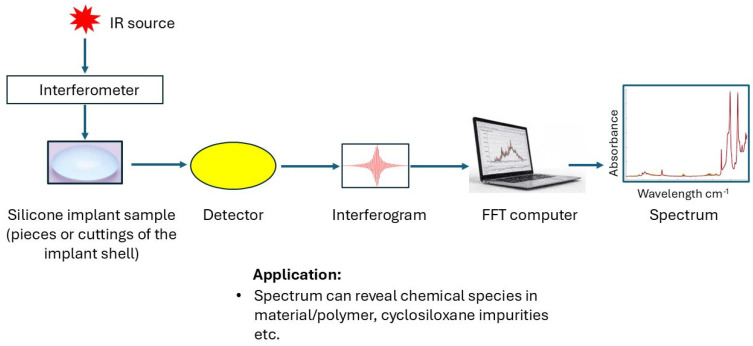
Schematic representation of the analysis of a silicone implant sample using an FTIR spectrometer. The IR source emits radiation that passes through an interferometer to encode the absorption data, interacts with a silicone implant sample, and is captured by a detector as an interferogram. The FFT computer processes the interferogram to produce a spectrum, revealing chemical properties such as vinyl signals or cyclosiloxane impurities. Fast Fourier Transform—FFT.

FTIR spectroscopy is an analytical technique used to characterize the chemical composition and structure of silicone breast implants, providing valuable insights into their biocompatibility [76]. FTIR is one of the fastest chemical methods used for the characterization of silicone implants. A major disadvantage of FTIR is that it mostly provides qualitative data on chemical functional groups. FTIR can also provide semi-quantitative data via the use of standards and estimation of peak intensities in a spectrum; however, the data output is not reliable for confirming that the concentrations are within the allowable ranges. This makes it a requirement to use FTIR with complementary methods during chemical characterization of mixtures and polymers. An FTIR spectrometer uses an interferometer to quantify the energy absorbed or transmitted by a sample. Infrared radiation emitted from the source reaches the interferometer, where spectral signal encoding occurs. The interferogram signal is either transmitted through or reflected from the sample surface, where specific energy wavelengths are absorbed (Figure 3) [52]. Subsequently, the beam passes through the detector and is conveyed to a processing computer for Fourier transformation into an absorbance/transmittance spectrum. Complex materials/mixtures or polymers can be difficult to study using FTIR as peak overlaps are difficult to separate and identify. FTIR can identify functional groups, analyze chemical bonds, and detect potential changes in the implant material over time. This is important for verifying the purity of PDMS/silicone implants, detecting both intended and unintended chemical modifications, and evaluating the potential degradation of polymers in the implants [6].

FTIR spectroscopy has been used in various studies to assess the chemical properties of silicone breast implants and their potential effects on biocompatibility. For instance, in a study examining different brands and types of silicone-based breast implants, FTIR was used with other spectroscopic techniques to distinguish implants based on their chemical characteristics [33,77]. In various studies on silicone breast implants, this technique has been used to analyze both the gel filling and outer shell, providing a deeper understanding of the chemical properties of the implants [29,77,78]. One study revealed that typical silicone-based implants show residual vinyl signals in the gel (from unreacted vinyl groups that remain from the manufacturing process) and that low levels of cyclosiloxane impurities are tolerable [33]. This information is critical for understanding the chemical composition of implants and their potential effects on their biocompatibility [29]. Kasalkoviova et al., 2024 utilized FTIR and EDS to analyze both native and modified PDMS implant surfaces and the elemental composition, respectively [66]. The authors exposed PDMS polymer to argon plasma pressure and then coated it with type I collagen, which led to enhanced implant biocompatibility, as demonstrated by increased myoblast cell guidance in an in vitro setting. In another study, FTIR was used to characterize multilayer coatings of polydopamine, graphene oxide, and collagen on silicone implants [67]. This study demonstrated the ability of FTIR to verify the successful application of complex surface modifications to implant surfaces. FTIR analysis has also been used to characterize a novel silicone implant material modified with a carboxybetaine ester analog [68]. FTIR spectroscopy confirmed the incorporation of zwitterionic groups, and the resulting silicone implant material demonstrated enhanced biocompatibility by reducing protein adsorption and bacterial adhesion [68].

FTIR can also be used during breast implant analysis to identify and characterize different silicone materials used during implant manufacturing. Beretta and Malacco demonstrated that FTIR spectroscopy, along with other complementary techniques such as Raman and NMR spectroscopy, can distinguish between brands and types of silicone-based breast implants based on their chemical characteristics [33,58]. This capability is important for market surveillance studies and quality control, ensuring that implants meet regulatory standards and are free from potentially harmful impurities. Another study used FTIR to investigate the aging characteristics of silicone rubber samples in harsh environments, including high-altitude areas, salt fog, and acidic environments [79]. The results revealed that silicone rubber exposed to these conditions experienced degradation, with the main chain being cut off, a reduced polymerization degree, and decreased hydrophobic functional group content [79]. These findings highlight the importance of FTIR in assessing the long-term stability and potential impact on the biocompatibility of silicone implants under various environmental conditions. FTIR has also been used to confirm the covalent grafting of bioactive polymers onto implant surfaces to improve their biocompatibility [80]. In one study, poly(methacrylic acid) (PMAc) and poly(acrylic acid) (PAAc) were grafted onto poly(dimethyl siloxane) (PDMS) surfaces to reduce bacterial adhesion and biofilm formation, which are primary concerns in breast implants [80]. FTIR analysis, along with XPS, confirmed the successful grafting of these polymers, demonstrating the utility of this technique in verifying surface modifications to enhance biocompatibility [80].

FTIR was used to comprehensively investigate the chemical and mechanical properties of breast implants from five manufacturers, and FTIR was used to monitor changes in the chemical structure after degradation under acidic and basic conditions [81]. The study revealed that all samples demonstrated changes in chemical structure after degradation, emphasizing the importance of parameters such as the degree of crosslinking and chemical composition in material performance [81]. The application of FTIR highlights its role in evaluating the chemical stability of implants under various conditions, which is crucial for assessing their long-term biocompatibility. FTIR has also been used to investigate the degradation of implant shell materials over time, decoupling the biological aspects of the degradation process [82]. The study found that although the chemical properties of the material did not change after 12 weeks of degradation, there were significant changes in its mechanical properties [82]. This observation highlights the importance of combining FTIR analysis with other techniques to fully understand the complex relationship between chemical structure and material performance in the context of biocompatibility. In another study by Amoresano et al., FTIR spectroscopy was used as part of a multidisciplinary approach to analyze 16 different breast implants after explantation [83]. FTIR analysis has helped identify traces of organic and inorganic substances in implants, highlighting the potential for bioaccumulation and tissue contamination [83]. This study underscores the importance of continuous medical surveillance and monitoring of the aging of breast implants.

### 4.2. Raman Spectroscopy

**Figure 4:**
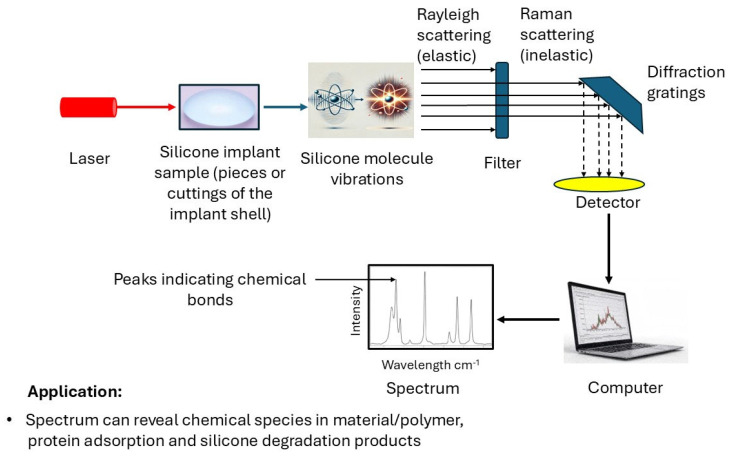
Schematic representation of the Raman Spectroscopy principle applied to silicone implants. A laser excites the silicone implant sample, inducing molecular vibrations that result in Rayleigh (elastic) and Raman (inelastic) scattering. A filter isolates the Raman scattering, which is separated by a diffraction grating and detected to produce a spectrum that reveals properties such as silicone oligomers or protein adsorption.

Raman spectroscopy is an important technique used to characterize the biocompatibility of silicone breast implants by providing detailed information about their chemical composition and structural properties. Raman spectroscopy is a vibrational spectroscopic technique often used in conjunction with FTIR analysis to yield additional insights into chemical composition and structure. This non-destructive analytical technique can detect subtle changes in the implant material that may affect its interaction with the biological tissues [84,85]. Raman spectroscopy works by measuring the inelastic scattering of monochromatic light when it interacts with molecular vibrations (Figure 4). Raman spectroscopy signals are often disrupted if a sample emits fluorescence after energy absorption. This issue is particularly pronounced if a sample contains organic or biological materials, as the emitted fluorescence can overshadow the weaker Raman peaks, complicating the accurate interpretation of the resulting spectra. Furthermore, Raman scattering is inherently weak, with only approximately one in a million photons contributing to the Raman signal. This low signal intensity frequently necessitates extended measurement durations or the use of highly sensitive and expensive equipment, particularly when analyzing trace components within a mixture. Raman spectroscopy also typically probes only the surface or near-surface layers of a sample due to its limited penetration depth. This characteristic can be disadvantageous when investigating the bulk composition of non-homogeneous mixtures or polymers, as deeper layers may not be adequately represented in the analysis of the bulk. The spectra generated by Raman spectroscopy can be complex, particularly for mixtures or polymers with multiple components. Overlapping peaks and varying scattering efficiencies further complicate the analysis, necessitating expertise and advanced software for effective data interpretation.

For silicone breast implants, Raman spectroscopy can identify specific chemical bonds and functional groups present in silicone materials [86]. This allows for the elucidation of the implant surface chemistry, detection of potential contaminants, and monitoring of changes that occur over time or due to interactions with the body. Beretta et al. used this technique to examine explanted PIP (Poly Implant Prothèse) implants, which were at the center of a major health scandal during which non-medical grade silicone was used [77]. Raman microscopy was utilized to analyze the chemical composition of the implant shell surface and was able to detect alterations in the silicone structure that might have occurred during the implantation period, providing insights into the material’s stability and potential degradation processes [76,77].

Raman spectroscopy can also be used to investigate the silicone gel filler inside breast implants. Near-infrared (NIR) spectroscopy, which is closely related to Raman spectroscopy, was used to analyze gel composition [77]. This analysis revealed information about the chemical structure, purity, and any changes that may have occurred due to interactions with body fluids or tissues. Another important aspect of implant biocompatibility that can be studied using Raman spectroscopy is protein adsorption on implant surfaces [87]. Protein adsorption is a critical factor in the body’s response to implanted materials, as it can influence cell adhesion, proliferation, the subsequent foreign body response, and thus biocompatibility [84,87]. Raman spectroscopy can detect and characterize proteins adsorbed onto the implant surface, providing valuable information about the implant-tissue interface.

This technique is also useful for studying biofilm formation on implant surfaces, which has been linked to complications such as capsular contracture and infection [88,89]. Raman spectroscopy can potentially identify bacterial species presence and their metabolic products, aiding the understanding of the mechanisms of biofilm formation and the development of strategies to prevent it. Raman spectroscopy can be utilized to verify the success of silicone implant modifications and monitor their stability over time [31,33]. Successful silicone implant modifications can be detected as altered chemical bonds or the deposition of new materials on the implant surface (coating), which will show as the appearance of new spectral peaks or shifts in the existing peaks. The ability of this technique to provide detailed chemical information makes it particularly valuable for studying novel coatings designed to enhance the biocompatibility of implants. For example, one study developed doxycycline-coated breast implants to reduce biofilm formation and surgical site infections [90,91]. Raman spectroscopy could potentially confirm the presence and distribution of such coatings.

### 4.3. Nuclear Magnetic Resonance (NMR) Spectroscopy

NMR spectroscopy is a powerful analytical technique for characterizing silicone breast implants and assessing their biocompatibility. This noninvasive method provides valuable insights into the chemical structure, composition, and potential degradation of implant materials, which are crucial factors in determining their long-term safety and performance. NMR spectroscopy can be used to analyze both silicone gel filling and the implant shell. For gels, liquid-state 1H and 13C NMR spectroscopy are commonly used to identify the chemical components and detect changes in the silicone structure over time [77,92]. These techniques can reveal the presence of low-molecular-weight siloxanes, such as D4, D5, and D6, which are potential breakdown products of silicone implants. In one study, the amounts of these cyclic siloxanes were below 100 ppm in explanted implants, suggesting minimal degradation in this study [77]. The implant shell can also be characterized by solid-state NMR spectroscopy. This analysis can help confirm the presence or absence of a barrier layer designed to prevent gel bleeding as a quality control process after implant manufacture. NMR analysis of explanted Poly Implant Prothèse (PIP) implants revealed that none of the examined envelopes had a barrier layer, potentially contributing to increased permeability [77].

One of the key advantages of NMR spectroscopy is its ability to detect and quantify mobile components, such as water, within implants. The presence of water in explanted implants may indicate the exchange of small molecules across the implant shell, even in visually intact implants [77,93]. This information allows for an understanding of the long-term behavior of implants in vivo and for assessing their potential for degradation or leakage. NMR can also be used to investigate the absorption of biological materials by the implants. In one study, proteins were detected in explanted implants, further demonstrating the bidirectional exchange of molecules between the implant and surrounding tissue [77,94]. This finding has important implications for biocompatibility, as it suggests that implants continue to interact with the host’s immune system over time. Further research into the bidirectional exchange of molecules between the implant and surrounding tissue is being undertaken to confirm whether this exchange is common to all implants. The use of advanced NMR techniques, such as high-temperature or high-pressure MAS NMR, has expanded the capabilities of this technique for studying breast implants under various conditions [95]. These advanced methods allow the observation of chemical interactions over a range of pressures and temperatures, simulating different physiological conditions [96]. This approach can provide valuable insights into the behavior of implant materials under stress and in response to environmental changes in the body.

NMR spectroscopy can be used in conjunction with other analytical techniques to comprehensively characterize the implant materials. For example, combining NMR with FTIR, XPS and thermogravimetric analysis can provide a detailed picture of the chemical and physical properties of implants [95]. The application of NMR spectroscopy in the analysis of silicone breast implants extends beyond surface characterization. This multi-analytical approach is particularly useful for studying modified implant materials, such as those functionalized with biocompatible coatings, to enhance their performance [97,98]. In the context of surface modification to improve biocompatibility, NMR plays a crucial role in confirming the successful attachment of functional groups and coating. For instance, 1H NMR can be used to verify the modification of hyaluronic acid (HA) or poly(ethylene glycol) (PEG) with dopamine groups, thereby enhancing the adhesion of biocompatible coatings to silicone surfaces [99].

Similarly to other spectroscopic techniques, NMR has some limitations. NMR spectroscopy exhibits lower sensitivity than other analytical techniques, such as mass spectrometry and infrared spectroscopy. NMR often encounters difficulties in detecting components of low abundance within mixtures or trace impurities. To mitigate these limitations, larger sample volumes or prolonged acquisition times are frequently necessary, which may be impractical for certain applications. The acquisition and maintenance of NMR equipment are associated with significant costs, and their operation requires specialized expertise. The necessity for cryogenic cooling, particularly for superconducting magnets, further contributes to the expense and complexity, rendering NMR less accessible for routine use or in smaller laboratories with constrained resources. In polymer analysis, the substantial molecular size and repetitive units often result in broad, overlapping peaks in the NMR spectra. This phenomenon diminishes spectral clarity, complicating the differentiation of similar structural features and the comprehensive resolution of complex polymer mixtures.

### 4.4. Gas Chromatography-Mass Spectrometry (GC-MS)

GC-MS is a sensitive analytical technique used to evaluate silicone breast implant compatibility and detect potential leachables, extractables, or degradation products. Coupling mass spectrometry with gas chromatography allows the separation of complex mixtures into individual components, which can then be identified by mass spectrometry. Chromatographic columns separate mixtures of volatile and semi-volatile leachables or extractables from the implants, while the mass spectrometer ionizes and fragments the molecules and separates them based on their mass-to-charge ratios (*m*/*z*) as they pass through a mass analyzer (e.g., quadrupole, time-of-flight (TOF), or Orbitrap) [100]. The resulting mass spectra were compared with available libraries or databases to identify the components of the mixtures. GC-MS is mostly utilized for the detection of volatile and semi-volatile compounds in devices such as implants and prostheses. This method allows for the detection and quantification of minute amounts of silicone compounds or degradation products that may leak from implants into the surrounding tissues or bloodstream [101]. A major disadvantage of GC-MS is its inability to resolve overlapping peaks or signals when analyzing complex materials and polymers. In addition, there is a high probability of sample degradation during sample preparation, which can introduce impurities that complicate the downstream analyses.

One study developed a highly sensitive large-volume injection-gas chromatography/mass spectrometry (LVI-GC/MS) method to detect siloxanes, which are key components of silicone breast implants, in blood samples [102]. This study focused on three cyclic siloxanes: octamethylcyclotetrasiloxane (D4), decamethylcyclopentasiloxane (D5), and dodecamethylcyclohexasiloxane (D6) [102]. By optimizing the extraction process using dichloromethane and implementing sample cooling during preparation, the study achieved extraction efficiencies of up to 100% and remarkably low detection limits of 0.03–0.05 ng of D4–D6 per gram of blood [102]. The GC-MS method demonstrated its utility in distinguishing between ruptured and intact silicone breast implants. Blood samples from patients with ruptured implants showed higher concentrations of D4 and D6, up to 0.57 ng D4/g blood and 0.16 ng D6/g blood, compared to those with intact implants [102]. The study established significant criteria for identifying ruptured implants based on D4 and D6 (concentrations above 0.18 ng D4/g blood and 0.10 ng D6/g blood). This analytical approach has proven to be more precise than traditional clinical diagnostic methods, such as mammary sonography, with an error rate of only 17% compared to 46% for sonography in a specific cohort [102].

The application of GC alone (although in most cases coupled with MS) to evaluate silicone breast implant compatibility extends beyond the detection of implant rupture. It can also be used to assess the long-term stability and potential degradation of implant materials [101,103]. By analyzing the chemical composition of implants or their extracts over time, scientists can identify any changes that may occur due to mechanical stress, exposure to bodily fluids, or age [101]. The compatibility of silicone breast implants is determined not only by their chemical stability but also by their interaction with the surrounding tissues. Gas chromatography coupled with MS can be used to analyze the compounds released by implants and assess their potential effects on the body.

Mass spectrometry alone (without chromatographic separation or other ionization methods) can also provide valuable molecular information for assessing the biocompatibility of silicone breast implants. Matrix-assisted laser desorption/ionization time-of-flight (MALDI-TOF) mass spectrometry imaging (MSI) has been used to investigate lipid adsorption on implant materials such as ultrahigh molecular weight polyethylene (PE-UHMW) (although it is less commonly used directly on silicone itself for this specific purpose) [104]. This technique allows for the visualization of the spatial distribution of lipids adsorbed on the implant surface. Additionally, mass spectrometry (typically LC-MS) can be used to analyze proteins adsorbed onto breast implant surfaces exposed to wound fluid [105]. Using an Orbitrap elite analyzer, 822 proteins bound to different implant surfaces were identified in a study [105]. This includes extracellular and intracellular proteins, with significant differences in binding affinities observed for inflammatory proteins, such as fibrinogen, and anti-inflammatory proteins, such as albumin [105]. The complement proteins C3, C5, and factor H also exhibited varying binding affinities across the implant types.

### 4.5. High-Performance Liquid Chromatography (HPLC) and Liquid Chromatography-Mass Spectrometry (LC-MS)

HPLC is a versatile technique used to separate, identify, and quantify the components of complex mixtures [106]. This capability could be valuable for analyzing the chemical composition of silicone breast implants (specifically extracts) or evaluating the potential leaching of non-volatile and semi-volatile compounds from implants. For instance, HPLC can be used to detect and measure additives (such as antioxidants or plasticizers, if present), impurities, and degradation products in silicone materials used in breast implants [107]. This type of analysis is important for assessing the purity and stability of implanted materials over time. One potential application could be the monitoring of the release of silicone compounds or other substances from implants into surrounding tissues or fluids. This could help assess the long-term safety and biocompatibility of implants. Coupling HPLC with other analytical techniques, such as FTIR spectrometry or MS, can provide complementary information about the chemical structure and interactions of implant materials. Developing and validating a specific HPLC method for this application would require extensive research and optimization tailored to the unique properties and expected analytes of silicone breast implants.

Advanced HPLC techniques, particularly ultra-performance liquid chromatography (UPLC), offer significant improvements over traditional HPLC methods in analyzing silicon breast implant extracts in terms of compatibility, including resolution, speed, and sensitivity. This improved resolution is important for detecting and separating potential contaminants or degradation products from silicone breast implants, providing a more comprehensive assessment of their compatibility with human tissues.

LC-MS shares foundational principles with GC-MS; however, it is particularly suited for examining compounds with lower volatilities. This category includes various substances, such as polymer additives, detergents, plasticizers, antioxidants, and higher-molecular-weight siloxanes. In LC-MS, the separation process relies on the interactions between the compounds and mobile and stationary phases. Typically, in reversed-phase LC, the mobile phase is a polar liquid, whereas the stationary phase consists of a nonpolar solid coating on the support material within the column. Once separated, the individual fractions are transferred to the mass spectrometer, where they are ionized, for example, via electrospray ionization (ESI). This process allows for the determination of mass-to-charge (*m*/*z*) ratios, which are crucial for the identification and quantification of compounds.

LC-MS is used to analyze complex matrices that include nonvolatile compounds. One of the main strengths of LC-MS is its ability to effectively separate mixtures of non-volatile substances, which are often complex. The success of liquid chromatography in LC-MS is largely due to the adaptability of non-volatile substances, which interact differently with the stationary phase depending on their polarity. Highly nonpolar compounds tend to be strongly retained by the stationary phase (in the reversed phase), whereas those with less retention move more quickly, leading to the highly efficient separation of complex mixtures. However, it is important to recognize that the analysis of LC-MS data, particularly when dealing with complex matrices, is difficult and time-consuming. This is true even for the extensive mass spectrometry data libraries currently available.

The main problem often encountered in LC-MS analysis is that other substances in the sample (matrix effects) can interfere with the ionization process of the mass spectrometer. This issue occurs more frequently with LC-MS than with GC-MS because of the sample introduction and ionization methods used. Interference can occur in different ways, such as ion enhancement, ion suppression and adduct formation. During ionization, substances eluted from the column simultaneously can change the ionization of the target analyte. For instance, high salt levels can block other substances from ionizing, thereby suppressing ionization. However, some organic solvents and surfactants can enhance the ionization of other substances (ion enhancement). Adduct formation, in which ionized compounds react with mobile phase components or other analytes, can make the mass spectrum more difficult to read by adding unexpected peaks or changing the *m*/*z* values. This makes it difficult to identify and measure the components of a mixture. Ionization interference can be a significant problem because it can lead to incorrect estimates of substance concentrations. Methods such as internal standards or matrix-matched calibration are often used to address these issues. These methods help adjust the data to account for matrix effects on ionization, making the measurements more accurate and reliable. These strategies are crucial for ensuring the accuracy and consistency of LC-MS results, particularly for complex samples such as those extracted from medical devices.

### 4.6. Summary and Comparison of Chemical Methods or Techniques Used to Analyze Silicone Implant Biocompatibility

Chemical methods/techniques for silicone implant biocompatibility characterization are often integrated for comprehensive profiling, as standalone methods may overlook subtle interactions in physiological environments. A comparison of the sensitivities, advantages, and limitations of various methods/techniques underscores the need for method complementarity to ensure robust biocompatibility evaluations, particularly as silicone implants evolve toward personalized and sustainable designs (Table 3). FTIR, including attenuated total reflectance (ATR-FTIR), detects functional groups such as Si-O-Si bonds with moderate sensitivity (1% *w*/*w* for impurities), making it suitable for bulk and surface analyses. NMR spectroscopy, particularly 29Si-NMR, offers higher sensitivity (0.1% *w*/*w*) for siloxane configurations and crosslinking density measurements. Raman spectroscopy, with sensitivity around 0.5% *w*/*w*, enables non-destructive mapping of oxidative changes. Compared to FTIR, Raman spectroscopy has superior spatial resolution (~1–2 μm vs. 50–100 μm for FTIR); however, FTIR excels in accessibility. The advantages of spectroscopic methods include rapid, label-free analysis; however, their limitations include the poor quantification of trace leachables and interference from complex matrices, such as biological fluids.

GC-MS achieves high sensitivity (<1 ng/g for volatiles like D4–D6 cyclosiloxanes). LC-MS targets non-volatiles with sub-ppm sensitivity. High-performance liquid chromatography (HPLC), often with UV detection, has moderate sensitivity (~ppm). GC-MS outperforms LC-MS for volatiles but requires derivatization for non-volatiles, whereas LC-MS offers broader analyte coverage. The advantages of this method include precise quantification and identification via mass spectra; however, its limitations include high costs, destructive sample preparation, and challenges associated with the low solubility of silicone.

Inductively coupled plasma mass spectrometry (ICP-MS) provides ultra-high sensitivity (<1 ng/L for elements such as platinum). XPS analyzes surface composition (0.1 atomic%). Energy-dispersive X-ray spectroscopy (EDS), often coupled with SEM, has a lower sensitivity (0.5% *w*/*w*) but enables the elemental mapping of the sample. ICP-MS surpasses XPS and EDS in trace detection but is destructive, while XPS offers surface specificity (depth ~10 nm) unattainable by bulk methods like ICP-MS. The advantages of this method include high precision for regulatory compliance (e.g., FDA thresholds for unknowns <1 ppm); however, its limitations include equipment expense and potential matrix effects in biological samples.

The integration of these methods enhances their overall reliability. Emerging approaches, such as time-of-flight secondary ion mass spectrometry (ToF-SIMS) for surface molecular imaging (~ppm sensitivity), address the limitations of spatial resolution. However, challenges persist as spectroscopic methods lack trace sensitivity, chromatographic techniques demand expertise, and elemental analyses overlook organic leachables. Future directions include the integration of AI for data fusion, as in the predictive modeling of toxicity from GC-MS datasets, to streamline the biocompatibility assessments of advanced nanocomposites.

## 5. Physical Methods for Characterization of Silicone Implants

**Figure 5:**
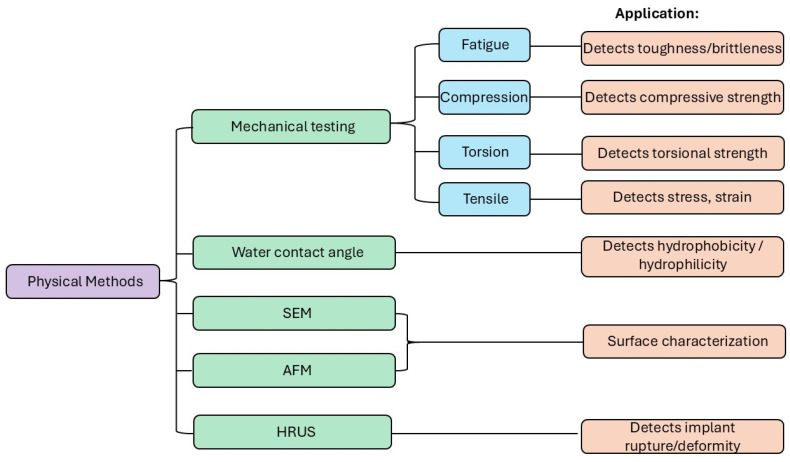
Physical Methods Commonly Used to Characterize Silicone Implant Properties. Surface characterization includes atomic force microscopy (AFM) (nanoscale roughness), scanning electron microscopy (SEM) (surface topography), and WCA (hydrophobicity/hydrophilicity). In vivo imaging uses HRUS (Implant Integrity). Mechanical testing includes tensile (stress/strain strength), torsional, compressive, and fatigue testing. Applications include scanning electron microscopy (SEM) for surface topography and biofilm detection, and high-resolution ultrasonography (HRUS) for rupture identification. AFM—Atomic force microscopy; SEM—scanning electron microscopy; WCA—water contact angle; HRUS—High resolution ultrasound.

Physical characterization focuses on the surface and mechanical properties of implants, which significantly influence their interaction with biological tissues. These properties determine cell adhesion, protein adsorption, and mechanical stability, all of which are critical for the biocompatibility of the material. A rigorous characterization program is required to determine the mechanical properties of implants to ensure that the required durability and performance are met. Physical characterization allows for the understanding of how the structural and mechanical properties of silicone implants affect interactions with tissues; however, it struggles to bridge the gap to clinical relevance without complementary biological context. Key physical methods include SEM, AFM, high-resolution ultrasound (HRUS), contact angle measurements, and tensile testing (Figure 5; Table 4).

### 5.1. Scanning Electron Microscopy (SEM)

**Figure 6:**
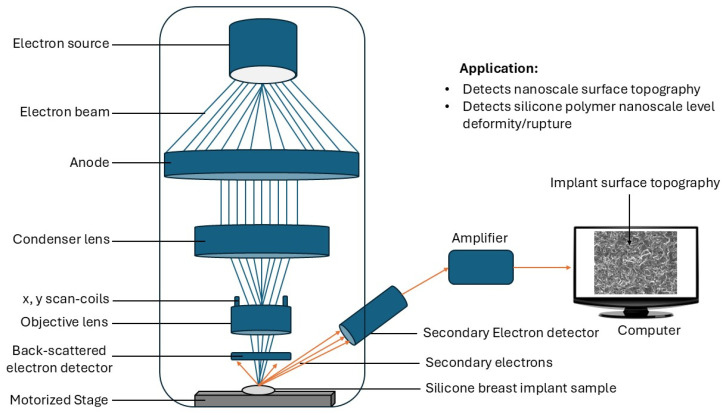
A scanning electron microscope projects a beam of electrons onto the sample surface. The electrons interact with the atoms in the sample, and the resulting secondary electrons are detected using a secondary electron detector. The secondary electrons passed through the amplifier and produced a high-resolution image of the sample surface at the nanoscale level.

SEM is an essential tool for characterizing surface properties and assessing the biocompatibility of silicone breast implants. A scanning electron microscope produces a beam of electrons from an electron source on the sample surface (Figure 6). The electrons interact with the sample atoms to produce signals or secondary electrons, which are detected by a secondary electron detector. The signal from the secondary electrons is used to produce high-resolution images of the implant surfaces and allows the examination of features relevant to cell-material interactions at the microscopic level. SEM has been used to analyze the surface topography and roughness of different silicone implants [11]. In a study by Barr et al., SEM was used to examine the surface characteristics of smooth, textured, and polyurethane-coated silicone implants [11]. The study showed that the surface roughness (Ra) values ranged from 0.2 ± 0.03 μm for smooth implants to 32 ± 7.0 μm for microtextured implants [11]. Various other studies have also utilized SEM to study the surface topography and roughness [121,122,123]. This information is important for understanding the influence of surface properties on cell adhesion and tissue integration. In a study by Atkins et al., SEM was used to observe the morphology and distribution of dermal fibroblasts cultured on different silicone rubber surfaces [124], demonstrating how SEM can provide valuable insights into cell-material interactions and their potential impact on the biocompatibility.

Another application of SEM in silicone implant research is the examination of protein adsorption and extracellular matrix (ECM) formation on implant surfaces after biological exposure. SEM can be utilized to study the formation and ultrastructure (revealing structure at the nanometer scale) of the capsular tissue surrounding silicone implants in animal models [125,126,127]. These studies revealed the organization and density of collagen fibers, thus providing insights into the mechanisms of capsular contracture. SEM has also been valuable in assessing the impact of various treatments on silicone implant biocompatibility. For example, Kuehlmann et al. (2021) implanted microtextured silicone implants in mice and harvested capsules on days 15, 30, and 90 post-implantation [128]. SEM revealed progressive fibrotic accumulation within the implant concavities, with histiocytic infiltration increasing over time and collagen fibers shifting from unidirectional to disorganized patterns by day 90 [128].

SEM operates under vacuum conditions, which can potentially alter or damage certain samples. This is particularly pertinent for implants or polymers, especially those containing biological components or volatile substances, such as those that are subjected to surface modifications. The vacuum environment may lead to moisture loss, outgassing, or structural alterations, thereby compromising the accuracy of surface property analysis, as the sample may no longer represent its natural state. Most polymers and implants are non-conductive, necessitating the application of a thin conductive coating, such as gold or carbon, to prevent charging under electron beams. However, this coating can obscure fine surface details or modify the surface chemistry, posing a significant limitation when the true surface composition is crucial for understanding material behavior. Although SEM provides excellent topographical information, it does not directly reveal the chemical composition of the surface of the sample. For implants and polymers, where surface chemistry often influences performance characteristics such as biocompatibility or adhesion, supplementary techniques such as EDS are required. This requirement adds complexity, time, and cost to analytical processes.

### 5.2. Atomic Force Microscopy (AFM)

AFM is a useful tool for characterizing the biocompatibility and relevant surface properties of silicone breast implants at the nanoscale. AFM offers unique capabilities for imaging and probing biological samples under various conditions, including aqueous conditions and nanometer-scale resolution. This is particularly valuable for studying silicone breast implants, as it allows for the examination of the implant surface topography and mechanics in a physiologically relevant environment. High-resolution imaging can reveal the surface topography, roughness, and potential defects that may affect biocompatibility [129,130]. One of the key advantages of AFM in studying implant biocompatibility is its ability to simultaneously acquire structural and mechanical information regarding the sample surface [129,131]. This is important for understanding how the surface of the implant interacts with surrounding tissues and cells.

Contact or tapping mode AFM can be used to obtain high-resolution topographical images of the implant surface, revealing micro- or nanoscale features that may influence cell adhesion or protein adsorption [110,120,132,133]. The Peak Force Tapping (PFT) mode, which is a recent advancement in AFM technology, allows simultaneous imaging and mechanical property mapping of the sample surface [134]. This could be particularly useful for examining changes in implant surface properties over time or in response to different physiological conditions. In addition, force spectroscopy measurements can provide valuable information regarding the mechanical properties of the implant surface, such as elasticity and adhesion forces, at the nanoscale level [35,135]. The ability of AFM to operate in liquid environments is especially relevant for studying silicone breast implant biocompatibility, as it allows for examination of the implant surface under physiological conditions, potentially revealing how surface properties change in the presence of biological fluids or cellular components [136,137,138]. This could provide insights into protein adsorption, cell adhesion, and biofilm formation on implant surfaces [139].

An interesting application of AFM in studying implant biocompatibility is the use of functionalized tips to probe specific interactions between the implant surface and biomolecules or cells [140]. By modifying the AFM tip with relevant proteins or cell membrane components, researchers can directly measure the adhesion forces and binding affinities between the implant surface and biological entities. This could provide valuable insights into the molecular mechanisms underlying implant integration and rejection [138]. However, it is important to note that AFM measurements, especially those of biological samples, can be challenging and are prone to artifacts if not performed carefully. AFM operates by scanning surfaces on a point-by-point basis, rendering it a relatively slow technique, particularly when high-resolution imaging is required. This time-intensive nature poses a significant limitation in contexts such as implants or polymers, where assessing surface uniformity or identifying defects over extensive areas is crucial. Consequently, the analysis of multiple samples or large surface regions becomes impractical, potentially impeding research progress and quality control processes. AFM requires a highly stable environment to obtain accurate results. External disturbances, such as vibrations, temperature fluctuations, or other environmental factors, can introduce errors or artifacts into the measurements. This requirement for a controlled laboratory setting may not always be feasible in industrial or clinical environments where implants are produced or tested, thereby restricting its practical application outside specialized facilities. In most cases, AFM is not used as a standalone technique for the characterization of mixtures or polymers, requiring the use of complementary techniques such as spectroscopy or mass spectrometry, thereby increasing the complexity and cost of the characterization process.

### 5.3. Contact Angle Measurements/Optical Tensiometry

**Figure 7:**
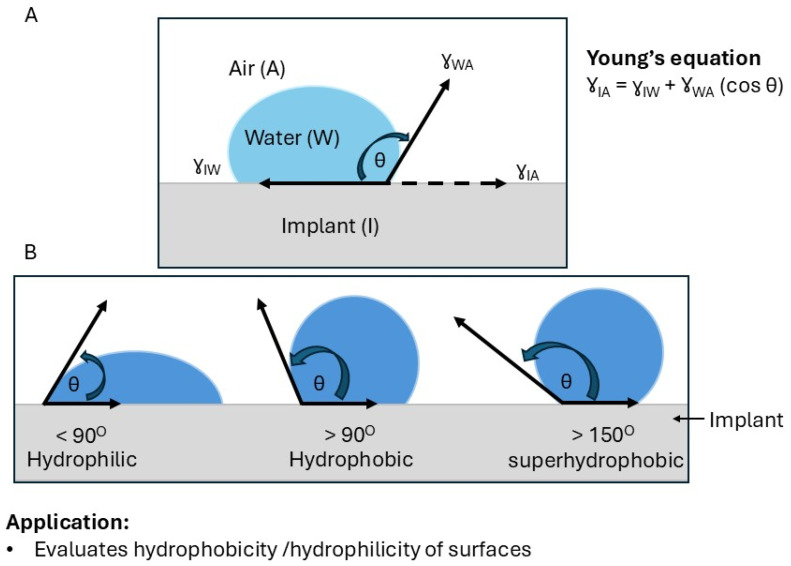
Schematic representation of the water contact angle (WCA) measurement on silicone implant surfaces. (**A**) Water drop on a silicone implant surface in thermodynamic equilibrium, with contact angle θ measured using an automatic goniometer. The interfacial tensions (γ_WA_, γ-_IW_, and γ_IA_) are related by Young’s equation (γ_IA_ = γ_IW_ + γ-_WA_ (cos θ)). (**B**) Hydrophilicity and hydrophobicity are determined by θ: θ < 90° (hydrophilic), θ > 90° (hydrophobic), and θ > 150° (superhydrophobic).

Water contact angle measurements are widely used to characterize the surface properties of silicone breast implants, via assessing the wettability (hydrophobicity or hydrophilicity) of the implant surface [141,142]. Wettability plays an important role in determining how the implant interacts with surrounding tissues and biological fluids. The water contact angle is the angle formed between a liquid droplet and a solid surface at the point of contact (Figure 7) [142]. For silicone breast implants, a higher contact angle (>90°) indicates a more hydrophobic surface, whereas a lower angle (<90°) suggests a more hydrophilic surface (Figure 7) [143]. This information is critical because the surface properties of an implant can significantly influence its biocompatibility, including bacterial adhesion, protein adsorption, and cell attachment [80,144]. For example, studies have shown that the hydrophobicity of poly(dimethylsiloxane) (PDMS) implants can promote bacterial adhesion and biofilm formation [117,145]. Water contact angle measurements are important for evaluating the modification of silicone implant surfaces. The effectiveness of silicone implant surface modifications can be assessed by measuring the resulting water contact angle [80,146].

A major limitation of water contact angle (WCA) measurements is that they only assess the outermost surface layer, typically within the top 0.5 to 1 nanometer. In the context of implants and polymers, where interactions with biological tissues or fluids often rely on both surface and subsurface properties, this shallow probing depth may prove insufficient. For example, in implants, long-term performance factors, such as degradation or protein adsorption, may be influenced by bulk or near-surface characteristics that WCA is unable to detect. Notably, the WCA technique is highly sensitive to surface impurities, including dust, oils, or chemical residues. Even minor contamination can significantly alter the contact angle, resulting in inconsistent and misleading outcomes. This issue is particularly challenging for polymers, which are prone to contamination during processing or handling, necessitating rigorous sample preparation to ensure reliable measurement. Furthermore, although the WCA provides a rapid assessment of wettability, it does not elucidate the reasons behind a surface’s hydrophobic or hydrophilic nature, as it does not offer direct information about surface chemistry (e.g., functional groups), which is critical for understanding material behavior.

### 5.4. High-Resolution Ultrasound

High-resolution ultrasound (HRUS) is a non-invasive imaging method capable of characterizing the implant status in vivo. While not directly measuring surface topography like SEM/AFM, it can differentiate implant types based on shell appearances and detect complications, aiding in identifying implant types without surgery and assessing their integrity. This is particularly relevant for conditions such as breast implant-associated anaplastic large cell lymphoma (BIA-ALCL) surveillance (detecting periprosthetic fluid) [147,148]. HRUS can effectively identify the type of implant and its manufacturer and detect potential complications such as rupture or fluid collection [149,150]. It is useful for screening silicone gel implants for silent ruptures, which are often asymptomatic but can lead to various health issues [147]. One study reported that 14% of women screened using HRUS had ruptured implants, of which 75% were surgically confirmed [147]. In conclusion, HRUS offers a convenient in-office alternative to magnetic resonance imaging (MRI) for certain aspects of breast implant screening [151]. It can help improve patient compliance with regular check-ups and provide valuable information regarding implant integrity and indirectly, some aspects potentially related to biocompatibility (for example, excessive fluid). However, further research is needed to establish standardized protocols and improve the accuracy of HRUS in characterizing all aspects of silicone breast implant biocompatibility [146,147].

### 5.5. Mechanical Methods for Characterization of Silicone Implant Biocompatibility

Mechanical testing methods (including tensile, fatigue, compression, torsion, shear, and hardness testing) are pivotal for assessing the biocompatibility of silicone breast implants, as they evaluate material integrity under physiological stresses, preventing failures such as rupture or degradation that could release silicone particles, triggering inflammatory responses, capsular contracture, or systemic toxicity. These tests align with the ISO 10993 and ISO 14607 standards [2,24], ensuring that implants mimic tissue biomechanics to minimize adverse biological interactions.

Tensile testing is an important method for characterizing the mechanical properties and durability relevant to biocompatibility of silicone breast implants. This test is performed by applying a controlled tensile force to a material sample and observing its behavior, thus providing valuable information regarding its strength, elasticity, and durability, as per ASTM D412/ISO 37 [110,152,153,154,155]. In one study, tensile and compressive tests revealed variations in the mechanical characteristics during silicone implant aging, affecting the stiffness, deformability, and strength of the implants [156]. Another study conducted tensile tests on up to 110 dumbbell-shaped specimens per implant, allowing for detailed mapping of the mechanical properties of the silicone shell [157]. This approach provides novel insights into the variation in the mechanical behavior across the implant surface. The study also proposed a map that clearly showed separated clusters for different manufacturers and product categories, highlighting the importance of tensile testing in comparing different implant types [157].

Fatigue testing simulates cyclic loads, for example, 6.5 × 10^6^ cycles at 3.3 Hz per FDA guidance, in assessing endurance against repetitive body movements [16]. Fatigue testing predicts longevity, with gel-filled implants failing via shell cracks, leading to gel leakage and immune activation. Structured implants exhibit lower rates (2.1% vs. 7–13%) of fatigue failure, thus demonstrating enhanced biocompatibility [16]. Compression testing measures resistance to loads (for example, up to 6000 N) and evaluates shape retention and contracture risk per ASTM D695 [16,158]. Torsion testing assesses the shear modulus and strength under twisting forces, according to torsion standards, adapted for implants to evaluate rotational stability in dynamic tissues. A good and biocompatible silicone implant would resist torsional stresses, averting delamination and particle release, which could elicit foreign body responses. Shear testing applies lateral forces (for example, 40 mm displacement over 2 × 10^6^ cycles) as per ISO 14607 and gauges interfacial integrity. This is critical for preventing the migration and chronic inflammation of breast implants. Hardness testing using Shore A or durometers quantifies firmness and correlates with palpability, with overfilled implants showing linear increases. Hardness testing optimizes the softness of the implant to reduce capsular fibrosis [159]. Collectively, these mechanical tests or methods help in the design of biocompatible silicone implants and can help minimize complications, such as granulomas or autoimmune issues, by ensuring mechanical stability.

### 5.6. Summary and Comparison of Physical Methods/Techniques Used to Analyze Silicone Implant Biocompatibility

A comparison of the sensitivities (e.g., resolution or detection limits), advantages, and limitations of the physical methods/techniques used for silicone implant biocompatibility characterization is presented (Table 5). The major limitations of physical methods and techniques include sample size constraints and the inability to capture biological interfaces. Other common limitations include the need for vacuum requirements for SEM/AFM and sensitivity to artifacts from sample preparation. The major limitations of dynamic mechanical thermal analysis (DMTA-DSC/TGA) include overlooking mechanical synergies, while aging extrapolates imperfectly to human variability. The integration of physical methods and techniques yields holistic insights. Challenges include method complementarity gaps, as mechanical tests ignore nanoscale effects, and the ethical need for nondestructive alternatives. Emerging tools, such as nanoindentation (~nN force sensitivity) and in situ thermal-mechanical testing, promise advancements, particularly for nanocomposites. AI-driven fusion of datasets can predict silicone implant failures by correlating surface metrics with clinical outcomes.

## 6. Biological Methods for Characterization of Silicone Implants

**Figure 8:**
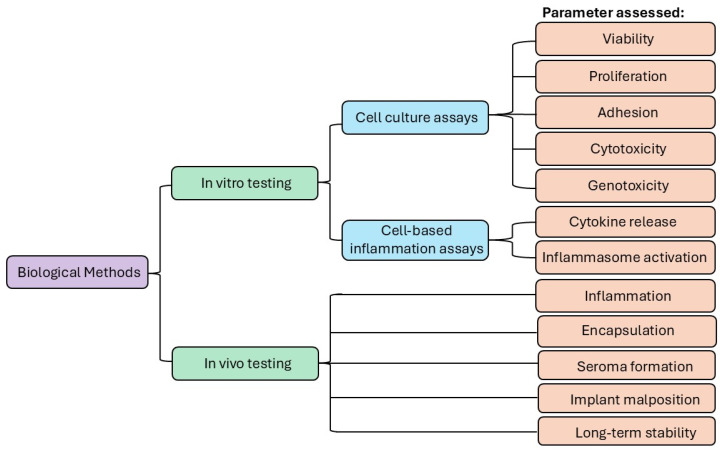
Biological methods are commonly used to evaluate the biocompatibility of silicone implants. In vitro testing includes inflammation assays and cell culture assays. In vivo implantation is used to evaluate inflammation, encapsulation (periprosthetic tissue formation), long-term stability, seroma formation, implant dislocation, and histological analysis.

Biological methods are essential for assessing the interaction of silicone implants with living tissues and provide insights into their biocompatibility through direct testing with biological systems. These methods can be divided into in vitro and in vivo approaches (Figure 8). In vitro biological methods can be further divided into cell culture (adhesion, cytotoxicity, proliferation, etc.) and inflammation assays.

### 6.1. In Vitro Testing

#### 6.1.1. Cell Culture Tests

Cell culture tests play an important role in assessing the biocompatibility of silicone implants and provide valuable insights into how silicone implants interact with living tissues and bodily fluids in controlled laboratory settings. These tests offer a controlled environment for evaluating the potential effects of silicone implants on cellular behavior, growth, and function. Cell culture tests for silicone implant biocompatibility can be performed using various cells and advanced in vitro models such as 3D skin equivalents [42]. The versatility of in vitro tests lies in their ability to use various cell types, including fibroblasts, macrophages, and epithelial cells, to mimic the different tissues with which silicone implants may come into contact (Table 6). These tests are advantageous because of their relatively low cost, high throughput, and capacity to isolate specific cellular responses without the complexity of whole-organism studies. However, these models have limitations, including their inability to fully replicate complex physiological environments such as systemic immune responses or long-term tissue interactions. Despite this, in vitro methods are widely endorsed by international standards, such as ISO 10993, which guides the biological evaluation of medical devices, including specific protocols for cytotoxicity testing (ISO 10993-5) [26,27,39]. The ISO 10993-5 biocompatibility strategy categorizes tests according to the implant nature of contact with body tissue or fluid, and their duration in the body. This section outlines the recommended approaches for cytotoxicity testing. Therefore, ISO 10993 is the primary standard used by scientists and manufacturers for biocompatibility evaluation. Advanced in vitro models that closely mimic human skin can provide a more accurate representation of the implant-tissue interface than traditional 2D cell cultures. In a study by Nuwayhid [42], scientists established a co-culture of keratinocytes and fibroblasts to study how a 3D ‘skin equivalent’ interacts with a breast implant surface [26,27,39]. To produce the 3D ‘skin equivalent’ with an intradermal implant, the authors added circular pieces of a smooth silicone breast implant cut from the implant shell to the co-culture of cells in 12-well plates. The data from this model demonstrated that a four-layered epidermis and a dermal component closely resembled native human skin, and the silicone implant samples did not cause cell death but triggered an inflammatory cytokine response [42]. The presence of tight junctions, an important feature of the skin barrier, was confirmed using immunofluorescence [42].

When assessing the biocompatibility of silicone implants using biological methods, the focus is on various key aspects, including cell adhesion, cell differentiation, cytotoxicity, and inflammatory response [16,42]. Cell attachment and proliferation assays are commonly used to investigate biological responses to implants [112,166]. These assays typically use fibroblasts and macrophages, which are relevant for assessing the integration or reaction to silicone implants (Table 6). Cell adhesion, or the strength of cell attachment to a silicone implant surface, can be evaluated by performing enzymatic trypsin detachment. In this assay, cultured cells are detached using trypsin at specific time points, and the detached cells are counted using cytometry or microscopy [73,105]. Detached cells were expressed as percentages of the original cell number. The fewer the detached cells at any given time, the stronger the attachment/affinity to the silicone-implant surface. This method has been used to achieve favorable surface biocompatibility in the past. Various studies have investigated the adhesion and proliferation of human fibroblasts on silicone substrates with varying topographies and have shown that fibroblast behavior is influenced by implant roughness and topography [167,168]. Using scanning electron microscopy and cell counting, one study revealed that fibroblasts exhibited enhanced attachment and spreading on microgrooved surfaces compared with that on smooth surfaces [165,168].

Cytotoxicity testing is a key step in determining whether silicone implants are toxic or release substances that could harm cells, which is critical for ensuring implant safety. These assays measure cell viability, metabolic activity, and membrane integrity after direct contact with the material, exposure to silicone extracts, and direct contact with the material. Cytotoxicity is typically evaluated using the MTT assay, live-dead staining, Annexin V apoptosis assay, and lactate dehydrogenase (LDH) assay, which measures cell survival and death [162,169,170]. The MTT assay, which quantifies the reduction in tetrazolium dye to formazan by metabolically active cells, is widely used owing to its sensitivity and simplicity [164,171]. In most studies, the MTT assay revealed that silicone implants did not significantly reduce viability compared to negative controls, indicating low cytotoxicity in vitro [164,171]. This aligns with the ISO 10993-5 criteria, which classifies a material as non-cytotoxic if the cell viability exceeds 70% of that of the control. In one study, human lens epithelial cells (HLEC) were exposed to silicone surfaces treated with different concentrations of toxic substances [162]. Cell viability (adherent and non-adherent) was evaluated via several assays including annexin V apoptosis assay as well as being stained by ethidium homodimer, and calcein [162]. This method proved to be sensitive in detecting implant toxicity, showing significant differences between treated and untreated silicone surfaces [162].

A study comparing various textured silicone gel breast implants (nanotextured, microtextured, and silicone foam) used both in vitro and in vivo approaches to assess cytotoxicity, inflammatory responses, and angiogenesis. Murine fibroblast cells (L929) were cultured in vitro on silicone membranes with different textures. Cell viability was assessed using the Alamar Blue assay, which showed higher viability of cells cultured on nanotextured silicone surfaces, whereas the MTT assay showed higher cell death in cells cultured on silicone foam after short-term exposure [164]. In another study, the integration of silicone samples into a 3D skin equivalent did not cause increased cell death, demonstrating that silicone implants were non-cytotoxic in that model [42]. Another approach to evaluate cytotoxicity is to use the lactate dehydrogenase (LDH) release assay, which detects cell membrane damage by measuring LDH leakage into the culture medium [42]. Various studies have utilized this method to evaluate the cytotoxicity of silicone or its derivatives on fibroblasts, and the results showed no significant increase in LDH release compared with the controls, reinforcing the conclusion that silicone gel and implants exhibit minimal cytotoxicity in these assays [170]. These studies highlight how in vitro cytotoxicity assays provide a rapid and standardized means of screening silicone implants for potential toxic effects, forming a foundational step in biocompatibility assessment.

Ensuring that silicone implants do not cause DNA damage or oxidative stress is essential for biocompatibility, as these effects can lead to mutations, cancer, and cellular dysfunctions. Genotoxicity is commonly assessed using the comet assay, which detects DNA strand breaks in individual cells [172]. Various studies have used the comet assay to evaluate the genotoxic potential of leachables from silicone implants in human lymphocytes and leukocytes [61,173,174]. The results of these studies showed no significant increase in DNA damage compared to the controls, suggesting that the tested silicone materials were non-genotoxic. This is a reassuring finding, given the historical concerns regarding silicone implants and their potential links to systemic health issues.

Oxidative stress, driven by the production of reactive oxygen species (ROS), is another critical parameter, as excessive ROS production can damage cellular components [175,176]. The dichlorofluorescein (DCF) assay is a standard method for detecting ROS in cells [177,178]. Studies have used this assay to investigate ROS production in fibroblasts exposed to silicone extracts [177,179]. They found no significant ROS generation, indicating that the tested silicone materials posed a low risk of inducing oxidative stress under these conditions. Together, these studies illustrate how in vitro tests can help screen for genotoxic and oxidative hazards, thereby providing a more complete picture of the safety of silicone implants in humans. While these assays are reductionist, they offer a controlled means to screen for effects that might be more difficult to detect in complex in vivo systems.

A few studies have been conducted using stem cells or a combination of cells to evaluate the biocompatibility of silicone implants. There is a need to consider more diverse cell types in biocompatibility testing [180], as this provides a more holistic understanding of how silicone implants interact with various tissues [181,182]. Although fibroblasts and keratinocytes are commonly used in in vitro assays and skin models, other cell types are also used depending on the intended application of the implant [183,184]. Various studies have used mammary fibroblasts and macrophages to study the interactions between silicone breast implants and breast tissue [182,185,186,187]. However, there is growing interest in using stem cells for these tests because they can provide insights into how implants affect tissue regeneration, wound healing and differentiation [188].

The choice of culture medium for biocompatibility testing is another important factor that can influence the results [16,181,189]. Most corrosion tests for biomedical silicone implants/materials are performed in simplified solutions (e.g., saline), which may not accurately represent complex biological environments. The Roswell Park Memorial Institute Medium (RPMI-1640) and DMEM culture media offer more realistic alternatives [190]. However, it is important to note that the medium can interact with the implant material. Research has shown that organic compounds in RPMI-1640 can form an electrochemically active adsorbed layer on material surfaces and that inorganic salt precipitation can be promoted by organic species [191]. These findings underscore the importance of using appropriate and well-characterized cell culture media and extraction vehicles to obtain reliable results on biocompatibility.

In a study investigating the dissolution of planar silicone thin films and micropatterned silicone pillar arrays in cell culture media, it was observed that the medium significantly accelerated degradation [192,193]. The silicone pillar arrays exhibited more prominent degradation effects, creating rougher surfaces and more complex surface states [194]. Despite these changes, in vitro cell culture studies have demonstrated the desirable biocompatibility of corroded silicone pillars, suggesting that even as the material degrades, it may maintain favorable interactions with cells in specific contexts.

#### 6.1.2. Cell-Based Inflammation Assays—In Vitro Testing

Inflammation assays play a key role in evaluating implant biocompatibility [195,196]. These assays help both manufacturers and scientists understand how the implant material interacts with the body’s immune system and its potential to elicit an inflammatory response. In vitro inflammation assays typically measure the release of inflammatory cytokines, such as interleukin-1β (IL-1β), IL-6, and IL-8, by cells in contact with the silicone implant surface or the activation of inflammasomes and multiprotein complexes that amplify inflammatory signaling (Table 7) [162,197]. The primary methods include enzyme-linked immunosorbent assays (ELISA) to quantify cytokine release and specific inflammasome assays to detect the activation of pathways such as NLRP3, which regulates IL-1β production. These approaches align with the principles outlined in ISO 10993-5 and other relevant sections of the biological evaluation of medical devices protocol. One of the earliest investigations by Kim et al. (2021) explored macrophage polarization in response to IL-4-coated silicone breast implants [131]. Using RAW 264.7 macrophages and ELISA, the authors quantified IL-6, TNF-α, and IL-10 release and found that IL-4 coating reduced pro-inflammatory cytokines (IL-6 and TNF-α) while increasing anti-inflammatory IL-10 production [131]. This modulation suggests a potential strategy for mitigating inflammation through surface functionalization, highlighting the adaptability of in vitro models for evaluating therapeutic interventions. Barthes et al. (2021) investigated the immunomodulatory effects of a cytokine cocktail (IL-10 and prostaglandin E2) released from hydrogels on 3D-printed PDMS implants [71]. The study showed, via ELISA data, reduced TNF-α and IL-6 levels and elevated IL-10 levels in macrophages, supporting the feasibility of preconditioning implants or using coatings to favor an anti-inflammatory phenotype [71]. Advanced 3D skin equivalents can be used to investigate the pro-inflammatory properties of silicone implants [42,198]. These models, consisting of co-cultured keratinocytes and fibroblasts, provide a more accurate representation of human skin than traditional 2D cell culture models [199,200]. In one study, silicone implant samples were integrated into these 3D skin equivalents, and the inflammatory response was evaluated using ELISA to measure cytokine release [42]. The results showed that silicone is not toxic to cells but can induce a pro-inflammatory effect, potentially linked to clinical observations of implant-associated irritation or fibrosis [42].

In addition to cytokine release, inflammasome activation is another mechanism by which silicone implants may exacerbate inflammation, mainly through degradation products such as wear debris and leached components. Meraz et al. (2012) investigated silicone microparticles (PDMS-derived) in THP-1 macrophages, using inflammasome assays to detect IL-1β release [201]. Their findings revealed a size-dependent effect, with particles smaller than 10 μm triggering significant IL-1β production via NLRP3 inflammasome activation [201]. The suppression of pro-inflammatory cytokines via IL-4 or IL-10 coatings points to surface modification as a viable strategy to reduce adverse reactions, potentially decreasing capsular contracture rates. The role of leachables suggests stricter quality control of manufacturing additives, whereas broad cytokine responses in skin models call for tissue-specific testing where relevant.

**Table 7:** Studies conducted involving inflammation-based assays (in vitro characterization methods) for silicone implant biocompatibility assessment. These studies evaluated the levels of cytokine release and inflammasome activation in cells cultured on silicone implants or PDMS.

Aim of Study	Method or In Vitro Test	Cell Type	Silicone Implant Type	Cell Function Tested	Reference
Assess pro-inflammatory properties of silicone in a 3D skin equivalent	ELISA (IL-1α, IL-6, IL-8, IL-33, MCP-1, TNF-α)	Keratinocytes, fibroblasts	Silicone breast implant	Cytokine release	[202]
Evaluate monocyte activation and cytokine release in response to silicone	ELISA (IL-1β, IL-6, TNF-α)	Human monocytes	PDMS	Cytokine release	[175]
Investigate immune response to medical-grade silicone in PBMCs	ELISA (TNF-α, IL-6)	Peripheral blood mononuclear cells (PBMC)	Silicone dermal filler	Cytokine release	[203]
Examine macrophage polarization and inflammatory response to IL-4-coated silicone	ELISA (IL-6, TNF-α, IL-10)	RAW 264.7 macrophages	Silicone breast implant	Cytokine release	[131]
Assess immunomodulatory effects of cytokine cocktail on PDMS implants	ELISA (TNF-α, IL-6, IL-10)	Macrophages	PDMS (3D-printed)	Cytokine release	[71]
Investigate silicone particle-induced inflammasome activation	Inflammasome assay (IL-1β release)	THP-1 macrophages	Silicone particles (PDMS)	Inflammasome activation	[204]
Assess cytokine response to silicone surfaces in human lens epithelial cells	Multiplex assay (IL-1β, IL-8, TNF-α, IL-6)	Human lens epithelial cells (HLEC)	Silicone surfaces	Cytokine release	[162]

In vitro models have limitations because they lack systemic immune interactions, vascular dynamics, and long-term feedback loops that are present in vivo. For instance, while various studies have linked debris to inflammasome activation, the clinical relevance depends on the debris generation rates in patients, which in vitro studies cannot predict [205]. In addition, inflammasome can also be activated by silicone gel exposure after implant rupture [205]. Similarly, the anti-inflammatory effects of the coatings require validation in animal models to assess their durability and efficacy over time. Thus, these studies serve as critical screening tools and mechanistic probes, often guiding in vivo preclinical and clinical studies.

### 6.2. In Vivo Testing

#### Animal Implantation Studies

In vivo studies are important for assessing the biocompatibility of silicone implants by evaluating their interactions with host biological systems, including potential inflammation, encapsulation, and long-term stability within living organisms [206,207]. These studies revealed great insights into the host response and potential complications associated with implant use (Table 8). Inflammation is a key aspect of the body’s response to foreign materials, including silicone implant. In vivo studies mostly involve implanting silicone materials subcutaneously at relevant anatomical sites in animal models, such as rats, rabbits, and pigs, and monitoring the inflammatory response over time [207,208,209]. Various markers of inflammation, including the presence of inflammatory cells and the expression of pro-inflammatory cytokines, can be analyzed in surrounding tissues or systemically. In one study, round silicone implants were inserted into subcutaneous pockets on the dorsum of mice to evaluate capsule formation around the implants [210]. This approach has enabled scientists to study the host response to silicone implants in rat models. Periostin-deficient mice showed significantly lower numbers of inflammatory cells than wild-type mice, suggesting that periostin plays a role in the inflammatory response to silicone implants [210]. Doloff et al. examined the inflammatory response and capsular fibrosis triggered by miniaturized breast implants with different surface topographies inserted in the mammary fat pads of mice for up to one year and observed that surface topography mediates immune responses to the implants [121]. Barthes et al. evaluated the inflammatory response to the presence of 3D-printed PDMS tracheal implants coated with immunomodulatory hydrogels in mice over four weeks and revealed that the coating reduced pro-inflammatory cytokines and enhanced M2 macrophage polarization, thus improving implant biocompatibility in that model [71]. Yoo and co-workers studied how micro-texturing and multi-layer coatings affect PDMS implant biocompatibility by implanting PDMS disks subcutaneously in mice for 8 weeks [117]. The authors observed that the modified implants had thinner capsules and less inflammation than the smooth and uncoated controls [117].

Encapsulation, which leads to capsular contracture, is a common long-term complication associated with silicone implants [213]. In vivo studies have assessed the formation and characteristics of fibrous capsules around implants. Excessive fibrosis can lead to complications such as capsular contracture. Animal studies have been instrumental in investigating this phenomenon and exploring potential solutions to this problem. For instance, studies in periostin-deficient mice have demonstrated that periostin inhibition is critical for suppressing capsule formation on silicone implants after in vivo implantation [210]. This study demonstrated that periostin knockout mice developed significantly thinner capsules around silicone implants than wild-type mice, indicating a potential role for periostin in capsule formation [210]. These findings provide valuable insights into the mechanisms of capsule formation and potential strategies for improving implant biocompatibility. Other parameters measured included capsule thickness, collagen density, and the presence of specific cell types associated with fibrosis, such as fibroblasts and myofibroblasts, utilizing various techniques such as histological examination, immunohistochemical analysis, and biochemical characterization. For example, a study comparing different silicone breast implants in rats found that implants with smooth textures exhibited higher collagen formation in the capsule than the other samples tested [214]. Kang and co-workers examined how plasma treatment affects the biocompatibility of PDMS implants [215]. PDMS implants treated with plasma were subcutaneously implanted in rats, and tissue responses were observed after 2 and 4 weeks. Treated implants formed thinner, more adherent, and more vascularized fibrous capsules than untreated implants, suggesting better tissue integration [215]. PDMS implants with micropatterns coated with polylysine/hyaluronic acid were subcutaneously implanted in rats, and the results were observed after eight weeks [117]. Combining physical and chemical modifications appears to improve biocompatibility, with reduced pro-fibrotic markers and thinner capsules [117].

Long-term stability is another critical aspect of silicone implant biocompatibility. In vivo studies assess the integrity and performance of implants over extended periods, typically ranging from several weeks to months or years [97,216,217]. Scientists have evaluated factors such as implant dislocation, seroma formation, and the overall structural integrity of the implant. For instance, a study comparing two types of breast expanders in patients found that surface roughness significantly influenced fibrotic implant encapsulation, with smoother surfaces leading to improved biocompatibility and reduced capsule formation [218]. To comprehensively assess biocompatibility, a combination of histological, immunohistochemical, and molecular techniques is often used in studies. Histological analyses, such as hematoxylin and eosin staining and Masson’s trichrome staining, allow visualization of tissue structure and collagen deposition [214]. Immunohistochemical techniques were used to identify specific cell types and proteins associated with inflammation and fibrosis. For example, immunofluorescence staining has been used to detect markers such as α-smooth muscle actin (α-SMA) and vimentin to assess fibroblast activation (myofibroblast formation) [214]. Molecular techniques, including Western blotting and gene expression analysis, provide insights into the underlying mechanisms of host responses to silicone implants. Previous studies have examined the expression of connective tissue growth factor (CTGF), transforming growth factor-β (TGF-β), and vascular endothelial growth factor (VEGF) to understand the processes involved in capsule formation and inflammation [210].

Overall, in vivo testing is important for understanding the potential long-term effects of silicone implants on the local tissue environment, immune system, and overall health of the animal model, which can inform predictions in human patients. Although local tissue responses are important, it is equally critical to assess the potential impact of implants or their degradation products on distant organs and overall health, as indicated by the risk assessment. One study evaluated the effects of silicone implants on various animal organs and demonstrated that the implants were non-toxic to these organs under the study conditions [219]. These findings are essential for establishing the safety of silicone implants beyond the immediate implantation sites. The duration of animal implantation studies varies depending on the research objectives. Short-term studies may last for a few weeks, whereas long-term studies can extend for several months or years. For instance, one study examined the effects of implants in rats after 7, 14, and 30 days, whereas the same study in sheep was extended to six months [220]. This range of durations enables the observation of both acute and chronic responses to the silicone implants. In addition to traditional histological and biochemical analyses, advanced imaging techniques are often used in conjunction with animal implantation studies. These include scanning electron microscopy (SEM (of explanted tissues/implants), X-ray diffraction (XRD) (for material analysis), and immunofluorescence assays.

### 6.3. Summary and Comparison of Biological Methods or Techniques Used to Analyze Silicone Implant Biocompatibility

Biological evaluation anchors silicone implant biocompatibility assessment, evaluating host responses such as inflammation, cytotoxicity, and tissue integration to avert complications such as capsular contracture or implant-associated lymphoma. In vitro testing provides rapid and controlled assessments of cellular interactions, prioritizing cytotoxicity, hemocompatibility, genotoxicity, and inflammation (Table 9). Cytotoxicity assays such as MTT (3-(4,5-dimethylthiazol-2-yl)-2,5-diphenyltetrazolium bromide) measure metabolic activity, with high sensitivity (10% viability shifts). Hemocompatibility tests, including platelet adhesion via flow cytometry, with a sensitivity of approximately 1000 platelets/mm^2^, are used to evaluate the risk of thrombosis. Genotoxicity assays, such as the Ames test, detect mutagenicity (2-fold mutation rate), whereas comet assays quantify DNA damage (10% tail moment). Inflammation assays, such as cytokine ELISA (pg/mL sensitivity) for IL-6/TNF-α and macrophage polarization via RT-PCR, revealed FBR. The advantages of in vitro testing include ethical compliance, cost efficiency (approximately $1000–5000 per test), and scalability for screening surface modifications. The limitations of in vitro testing include oversimplification, with many assays lacking immune crosstalk or shear stress, which can lead to false-positive results.

In vivo models simulate holistic responses using animal implantation, histological analyses, and immunohistochemistry. Histology (H&E staining) detects cellular infiltration (1 cell/HPF), whereas immunohistochemistry (e.g., CD68 for macrophages) quantifies cell phenotypes (5% expression shift). Cytokine profiling in tissues (ng/g sensitivity) using multiplex arrays tracks chronic inflammation. Compared to in vitro studies, in vivo studies offer superior sensitivity to systemic effects but lower sensitivity to molecular details; however, histology can surpass in vitro assays in terms of spatial resolution. The advantages of in vivo testing include physiological fidelity and the ability to capture long-term fibrosis. The limitations of in vivo testing include ethical concerns, high costs (approximately $10,000–50,000), species variability (e.g., rats vs. humans in immune response), and poor predictability for rare events such as BIA-ALCL.

Advanced models, such as 3D equivalents and organ-on-a-chip systems, bridge the gap between in vitro and in vivo testing. Three-dimensional skin models, including the coculturing of fibroblasts/keratinocytes with silicone, are sensitive to cytokine surges of approximately 10 pg/mL IL-6, which mimics human skin thinning. Organ-on-a-chip systems simulate dynamic flow and detect shear-induced adhesion (0.1 dyne/cm^2^). The advantages of advanced models include their human relevance in the absence of animals and their ability to enable personalized in vitro testing. The limitations of advanced models include their complexity (sometimes requiring co-culture of cells), lack of standardization, and short-term viability (approximately 30 days).

Histological and genomic techniques have revolutionized our understanding of the host response to silicone breast implants, revealing the molecular underpinnings of foreign body response (FBR) and capsular contracture in human periprosthetic capsule samples. These methods enable temporal insights into immune activation and fibrosis progression, critical for implant longevity. Doloff and colleagues established that implant surface topography influences FBR across species, with smoother surfaces mitigating fibrosis in human capsules, primarily through histological and functional analyses [121]. The authors also used flow cytometry to identify periprosthetic immune cell tissues after mini-implant insertion in rats and rabbits [121]. Genomic studies include Mao and colleagues’ s RNA-seq analysis of 12 patient capsules which uncovered 93 differentially expressed genes enriched in lipid metabolism, with hub gene PRKAR2B correlating negatively with M1 macrophage and follicular helper T cell infiltration, suggesting metabolic-immune crosstalk in progression [221].

Wolfram and colleagues investigated capsule samples using high-throughput proteomics and delineated chronological biomarker changes in immune and fibrotic pathways, highlighting dynamic protein-level responses absent in pure transcriptomics [218]. In addition, Pluvy and colleagues combined histological analysis and gene expression profiling (RNA sequencing) of samples from human patients with silicone breast implants with different fillers (silicone or serum), surface topographies and/or shell rupture, and performed systematic cross-comparisons [205]. This study showed that exposure to silicone gel fillers, even in clinically asymptomatic cases, induced an immune response. This response includes the expression of markers associated with different autoimmune diseases. This study provides biological evidence of an association between silicone implants and autoimmune markers, highlighting the need for further research and stricter implant safety regulations [205].

Collectively, these omics approaches underscore a multifaceted, time-dependent host-implant dialogue that informs strategies to attenuate pathological FBR. Studies on human capsules allow for long-term biocompatibility analyses compared to in vitro/in vivo models and are far more representative of implant biocompatibility, although they are not easily accessible technically. Finally, in vitro/in vivo models do not fully recapitulate the human host microenvironment and may not accurately represent biocompatibility in general.

## 7. Integration of Biological, Chemical, and Physical Methods for Assessing Silicone Implant Biocompatibility

The integration of biological, chemical, and physical characterization methods provides a comprehensive approach for evaluating the biocompatibility of silicone implants. No single method provides a complete picture; thus, integration is crucial in this regard.

FTIR, Raman spectroscopy, SEM, NMR, contact angle measurements, and tensile testing can be integrated to provide a holistic understanding of the properties of implants and their potential interactions with biological systems [86,222]. For instance, although FTIR spectroscopy is a powerful tool for analyzing the chemical composition and structure of silicone breast implants, Raman spectroscopy can complement FTIR by detecting subtle changes in the silicone polymer backbone and side chains that may not be visible in FTIR spectra [33,86]. SEM is crucial for examining the surface morphology and topography of silicone breast implants [33]. Combining SEM imaging with EDS provides elemental composition maps of the surface. One study utilized Raman microscopy to analyze the implant envelope, near-infrared spectra, and GC-MS to examine the gel composition of explanted devices [77]. This highlights the importance of a multimodal approach to implant assessment that combines preclinical material chemical analysis with imaging techniques for a comprehensive evaluation across the product lifecycle.

AFM can also be combined with other complementary techniques to provide a more comprehensive understanding of the surface properties relevant to biocompatibility [117,129]. For example, integrating AFM with optical microscopy could allow scientists and researchers to correlate surface topography with cell behavior on implant surfaces [139]. Combining AFM with spectroscopic techniques, such as Raman or infrared spectroscopy (e.g., AFM-IR), can provide spatially resolved chemical information about the implant surface and adsorbed biomolecules [223,224].

Water contact angle measurements are frequently used along with other surface characterization techniques to provide a comprehensive understanding of implant biocompatibility [225,226]. For instance, XPS (for surface elemental composition and chemistry), FTIR (often ATR-FTIR for surfaces), and SEM are often used in conjunction with contact angle measurements to analyze surface chemistry, topography, and roughness [80,110,120,225]. Integrating these physical and chemical findings with biological data (e.g., cell adhesion and protein adsorption) allows the establishment of structure-property-function relationships that are crucial for assessing biocompatibility.

## 8. Structured Framework for Selecting Tests to Evaluate the Biocompatibility of Silicone Breast Implants

We propose a structured framework for selecting appropriate tests to assess the biocompatibility of breast implants, categorizing them based on their type: native/unmodified (textured or smooth) and modified implants.

**Figure 9:**
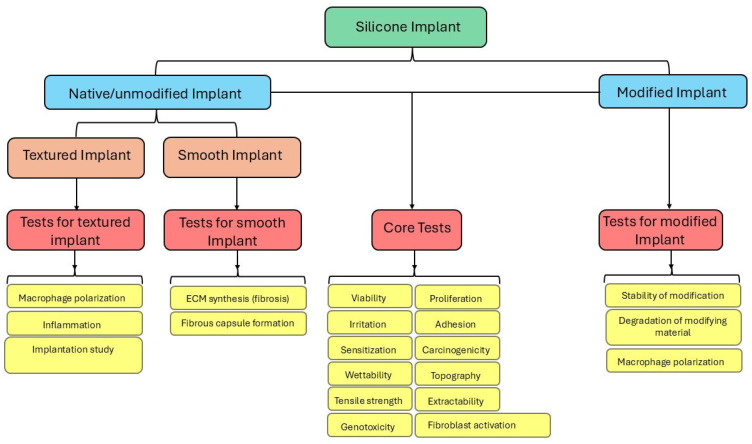
A suggested hierarchical approach starting with the implant type, branching into specific subcategories, and detailing core tests applicable to all implants, as well as tests that can be considered specific to textured, smooth, or modified implants. Ideally, all tests can be performed for all silicone implants, making this figure one of the first attempts at standardized test selection during silicone implant biocompatibility assessment. Colors are used to show the different levels of categories that can be used when deciding which tests to use.

Breast implants can be textured (largely removed from the market) or smooth, each with unique surface characteristics that interact differently with biological tissues, necessitating tailored biocompatibility evaluations. Figure 9 outlines a simplified hierarchical approach, starting with the implant type, branching into specific subcategories, and detailing the core tests applicable to all implants and tests that can be considered specific to textured, smooth, or modified implants. This suggested structured methodology can ensure that the evaluation process addresses both general biocompatibility concerns and risks specific to each implant type.

Central to the testing framework are essential assessments that are applicable to all breast implants, irrespective of their surface characteristics. These evaluations are designed to ascertain fundamental biocompatibility properties, ensuring that the implant does not elicit an adverse biological response. The core assessments include: proliferation: Evaluating whether the implant material supports or inhibits cellular growth is crucial for understanding tissue integration; adhesion: The extent to which cells adhere to the implant surface, which influences long-term stability and integration; viability: Determine the survival rate of cells in contact with the implant, ensuring that the material is non-toxic; cytotoxicity: To prevent tissue damage, it is essential to evaluate any toxic effects on cells; sensitization: It is crucial to assess the potential allergic reactions that could result in inflammation or rejection; irritation: It is necessary to determine whether the implant induces irritation of the surrounding tissues; genotoxicity: Evaluating the potential of an implant material to cause genetic mutations is a critical factor in assessing carcinogenic risk; carcinogenicity: Direct testing of the cancer-causing potential is imperative, given the long-term implantation of the device; wettability: Measuring the interaction of the surface with water is important because it influences protein adsorption and cell behavior; protein adsorption: Examining the interaction of proteins with the implant surface is vital, as it can affect immune responses; surface topography: Analyzing the physical characteristics of the surface is essential because they can influence biological interactions; tensile strength: Testing the mechanical durability of an implant is necessary to ensure its ability to withstand physiological stress; fatigue resistance: Assessing the implant’s capacity to endure repeated mechanical stress over time is crucial; extractable materials: Identifying any leachables or extractables from the implant that could pose a risk to the body is important. Fibroblast activation must be evaluated for both unmodified and modified silicone implants. These core tests provide a comprehensive baseline for biocompatibility and address the biological, mechanical, and chemical aspects of the performance of implants. They ensure that the implant is safe, does not provoke adverse reactions, and maintains its structural integrity over time.

Textured implants have a rough surface designed to promote tissue adhesion and reduce the risk of capsular contracture (It is important to note that although capsular contracture rates are reduced, it is also observed when textured implants are used), a common complication of scar tissue tightening around implants. However, this surface texture introduces unique biocompatibility concerns, particularly those related to immune responses and long-term tissue interactions. The two specific tests for textured implants were as follows: Macrophage Polarization: This test evaluates the response of macrophages (a type of immune cell) to textured surfaces. Macrophages can polarize into pro-inflammatory (M1) or anti-inflammatory (M2) states, and an excessive pro-inflammatory response can lead to chronic inflammation and implant rejection. Textured surfaces may exacerbate this response because of their roughness, which makes this test essential. Inflammation (ALCL Risk): Anaplastic Large Cell Lymphoma (ALCL) is a rare but serious cancer associated with textured breast implants, particularly those with higher surface roughness. This test assesses the inflammatory response that could contribute to ALCL, focusing on the role of the implant in triggering chronic inflammation, which is a known risk factor for this condition. The focus on ALCL reflects the growing awareness of this risk, particularly following reports linking textured implants to a higher incidence of lymphomas.

Smooth implants have polished surfaces that minimize tissue adhesion. While this reduces the risk of ALCL, smooth implants are more prone to complications such as capsular contracture due to their lack of tissue integration. Specific tests for smooth implants are as follows: ECM Synthesis (fibrosis), and Fibrous Capsule Formation (Capsular Risk), which evaluate the tendency of the implant to trigger fibroblast activation, leading to myofibroblast formation, ECM synthesis, and ultimately, fibrous capsule formation. Myofibroblasts are key players in wound healing but can contribute to excessive scar tissue formation around implants, resulting in capsular contracture. These tests are particularly relevant for smooth implants because their lack of surface texture often leads to poor tissue integration, which increases the risk of complications.

Modified implants (or coated) require additional surface treatment to enhance biocompatibility, reduce complications, or improve integration [6,7]. These coatings introduce new variables, such as coating degradation over time, which must be assessed. Figure 9 outlines two specific considerations for the modified implants. Core Tests: The core tests listed earlier (proliferation, adhesion, viability, etc.) are repeated for the modified implants to ensure that the coating does not alter the fundamental biocompatibility of the implants. This step is crucial because coatings can introduce new biological interactions that must be thoroughly evaluated. Degradation and Coating Stability: This test assesses the long-term stability of the coating by focusing on its degradation profiles. If a coating breaks down over time, it can release particles or chemicals into the body, potentially causing toxicity, inflammation, and other adverse reactions to the body. Ensuring the stability of the coating is essential for the long-term safety of the implant. Macrophage Polarization: This test evaluates macrophage response to modified surfaces.

## 9. Artificial Intelligence Integration in Silicone Implant Biocompatibility Characterization

The incorporation of artificial intelligence into the framework presents a robust mechanism for predicting the biocompatibility of silicone implants, thereby aligning with the objective of customizing the testing for specific applications. Machine learning algorithms trained on historical data derived from physical, chemical, and biological assessments can discern patterns and predict outcomes, such as cytotoxicity, irritation, and long-term degradation. For instance, supervised learning models may (in the future) forecast the behavior of a new silicone formulation in a permanent implant using data from analogous materials tested both in vitro and in vivo. This predictive capability can streamline testing efforts, minimize redundancy, and expedite development timelines.

Artificial intelligence facilitates sophisticated simulations of the interactions between implants and biological systems, thereby reducing the dependence on extensive animal testing. Techniques such as computational fluid dynamics (CFD) can be employed to model the fluid flow around implants, whereas finite element analysis (FEA) can be utilized to simulate the mechanical stress on adjacent tissues. Deep learning further augments these capabilities by analyzing histological images to identify subtle indicators of inflammation or fibrosis, which may differ based on the implant type or duration. Additionally, natural language processing (NLP) can extract information from unstructured data, such as adverse event reports and clinical notes, to detect early safety signals. These AI-driven methodologies can guide application-specific testing strategies, such as the chemical analysis of leachables for short-term implants or biological assays for long-term tissue integration.

Guidelines for data collection and sharing are essential for the effective implementation of AI within this framework. High-quality standardized datasets are the foundation of reliable AI models. Collaborative research initiatives should establish data-sharing platforms that balance patient privacy and research accessibility by employing standardized formats to ensure interoperability. For instance, data on implant surface properties, chemical composition, and biological responses should be consistently documented across studies to facilitate robust training of models. The framework can subsequently integrate AI outputs, such as risk scores or predicted failure modes, into decision-making processes, thereby indicating whether additional physical, chemical, or biological tests are necessary for a specific implant type and application.

Data quality and representativeness are paramount for AI. Biased or incomplete datasets that lack diversity in patient demographics or implant applications can lead to inaccurate predictions, thereby compromising the safety of patients. Collaborative efforts should prioritize the collection of comprehensive, high-quality data that reflect real-world scenarios. Ethical considerations, such as algorithmic bias and transparency, have also arisen. AI models must be validated to perform equally across populations, and their decision-making processes should be explained by the regulators and clinicians. Rigorous validation of real-world outcomes, facilitated by multicenter studies, is essential to ensure their reliability. For example, an AI model predicting implant-related inflammation can be tested in diverse patient cohorts to confirm its generalizability.

It is recommended that a centralized database for biocompatibility data be created that is accessible to stakeholders under the agreed protocols to support both collaborative analysis and AI training. It should also specify how AI predictions are fed into the decision-making process; for instance, using AI to recommend specific physical tests (e.g., tensile strength) for load-bearing implants or biological assays (e.g., sensitization) for long-term devices. Open-source AI tools should be encouraged to promote transparency and broader adoption.

### 9.1. Convolutional Neural Networks (CNNs) for Image Analysis

CNNs can be trained to analyze microscopic images (e.g., scanning electron microscopy or histological slides) of silicone implants. They can assess surface characteristics, detect biofilm formation, and evaluate tissue responses such as cell adhesion and proliferation. The benefit of this approach is that it enables the automation of image analysis, reduces manual effort, and provides consistent and objective measurement. This can help decision-makers quickly identify surface-related biocompatibility issues.

### 9.2. Machine Learning for Predictive Modeling

ML models can predict biocompatibility outcomes, such as the likelihood of inflammation or capsular contracture, using data from physical (e.g., surface roughness), chemical (e.g., composition), and biological tests. These predictions can guide testing priorities. This enables the early identification of high-risk implants, optimization of testing protocols, and supports data-driven decisions regarding which implants require further evaluation.

### 9.3. Natural Language Processing (NLP) for Data Integration

NLP can extract relevant information from the literature, adverse event databases, and clinical notes regarding silicone implants. This could inform the framework by highlighting known biocompatibility issues and emerging trends. This allows the decision-making process to be updated with the latest knowledge, reduces manual literature review time, and provides a broader evidence base for test planning.

### 9.4. AI-Driven Decision Support Systems

An AI decision support system can recommend specific biocompatibility tests based on implant characteristics (e.g., size and surface texture), intended use, and regulatory requirements. It can also flag potential areas of oversight in the literature. This streamlines the testing process, ensures compliance with standards, and helps researchers and manufacturers make informed and efficient decisions.

### 9.5. Deep Learning for Anatomical and Virtual Simulations

These models can simulate implant-tissue interactions or predict how surface modifications affect biological responses. For example, they may model the behavior of silicone implants under various physiological conditions. This reduces the reliance on extensive in vivo testing, lowers costs, and provides insights into implant performance before physical testing.

## 10. Challenges and Future Directions

Silicone implants, particularly silicone breast implants, have been widely used for cosmetic and reconstructive purposes since their introduction [6,227]. However, their use has been associated with various complications, including capsular contracture and BIA-ALCL [16,21,34]. To address these issues and improve implant biocompatibility, researchers have explored various biological, chemical, physical, and mechanical methods for characterizing and enhancing silicone breast implants.

Chemical characterization of silicone breast implants is important for understanding their long-term stability and potential for degradation. One of the principal challenges in chemical characterization is achieving comprehensive extraction of potential extractables and leachables from devices. Complex polymers may interact with solvents in unpredictable ways, resulting in incomplete extraction or material degradation, which complicates characterization. The identification of compounds using databases can be constrained by the size and comprehensiveness of the database, particularly in the evaluation of non-volatile organic compounds (NVOCs). Many potential degradation products and extractables from medical devices are not represented in these databases, rendering accurate compound identification difficult. Future research should focus on developing more sensitive and specific chemical analysis methods to detect the early signs of implant degradation and the potential leakage of silicone particles and other leachables.

Physical characterization of implants includes the assessment of surface properties such as roughness, wettability, and stiffness, which can significantly impact cell-material interactions and overall biocompatibility [12,16,21,76]. Advanced imaging techniques and surface analysis methods are required to better understand the relationship between surface properties and biological responses. Silicone implants often display non-uniform structures due to variations in polymer cross-linking and filler distribution. This variability poses challenges in accurately measuring physical properties such as elasticity, tensile strength, and surface texture across the implant. Evaluating changes in physical properties over time, such as wear, fatigue, or hardening resulting from biological interactions, is also difficult. Accurately replicating in vivo conditions in the laboratory to assess durability and stability requires complex setup and extended testing durations. Future research should focus on developing novel surface modification techniques to optimize these physical properties for enhanced biocompatibility. Future research should focus on developing more comprehensive mechanical testing protocols that better simulate in vivo conditions and stress distributions.

Biological methods for characterizing implant biocompatibility primarily focus on assessing foreign body responses and cell surface interactions. Recent advances in this area include modifications of the implant surface topography and chemistry to enhance protein adsorption and cell adhesion [13,16,21]. These modifications aim to improve implant integration and reduce the risk of complications associated with the procedure. However, challenges remain in developing standardized biological assays that can accurately predict in vivo performance. Assessing the biological response to silicone implants, including inflammation and tissue integration, is complex owing to the variability in patient immune responses. In vitro and in vivo models often fail to fully replicate human physiological conditions, complicating the prediction of long-term biocompatibility. Identifying and evaluating the biological impact of leachable compounds, such as low-molecular-weight siloxanes, in silicone implants is challenging. These compounds may induce subtle or long-term toxicological effects, necessitating sensitive assays and prolonged studies to detect adverse biological responses in humans to these compounds. Future research should involve the development of more complex in vitro models that can better mimic physiological environments and immune responses [16,21,34].

One of the main challenges in characterizing the biocompatibility of silicone breast implants is the lack of standardized testing methods across different regulatory systems and geographical regions [228]. This inconsistency makes it difficult to compare the results of different studies and establish universal safety standards beyond the baseline ISO 10993 requirement. Future efforts should aim to harmonize testing protocols and encourage the development of tailored frameworks, such as the one proposed in this study, to ensure consistent and relevant evaluation of implant biocompatibility worldwide. Advances in this area may include the development of 3D models, dynamic testing systems, and complex multicellular and potentially multi-organ systems to better predict implant performance and potential complications [207,229].

## 11. Conclusions

In conclusion, biocompatibility is a multifaceted and context-dependent concept that necessitates a range of assays to confirm the safe functionality of foreign materials such as silicone implants within the human body. Given the influence of various factors, manufacturers and scientists must assess the biocompatibility of materials by examining both the bulk and surface properties of silicone implants to understand and predict the body response (FBR). This review summarizes the commonly employed tests and assays that can assist both manufacturers and regulatory bodies in selecting the most appropriate tests and assays for silicone implants. However, it also highlights the limitations of generic approaches and advocates for tailored strategies. Future advancements to enhance the biocompatibility of medical devices include improvements in material characterization, creation of standardized application-specific testing protocols and frameworks, comprehensive long-term biocompatibility evaluations, incorporation of cutting-edge technologies (such as improved in vitro models and computational prediction), and addressing individual variability, whenever possible. These recommendations are intended to support researchers and industry professionals in expanding their understanding of biocompatibility, ultimately enhancing patient health and well-being, and fostering the development of safer and more effective medical devices. Careful design and characterization of surface properties via treatments or coatings, informed by detailed surface analysis and integrated biological testing, are essential for the development of medical devices that are not only functional but also safe and effective over their intended lifespan in the body. Ultimately, an integrated, risk-based, and application-specific approach to characterization is necessary to ensure silicone implants’ biocompatibility.

The suggested structured testing framework, as shown in Figure 9, reflects a comprehensive approach to biocompatibility evaluation, aligned with international standards such as ISO 10993, which provides guidelines for the biological evaluation of medical devices. By categorizing tests into core and specific assessments, the framework ensures that both general and type-specific risks are addressed, thereby providing a thorough understanding of the safety profiles of implants. For textured implants, the focus on macrophage polarization and ALCL risk highlights the need to balance the benefits of texturing (e.g., reduced capsular contracture) with the potential risks (e.g., chronic inflammation and lymphoma). For smooth implants, the emphasis on fibrous capsule formation underscores the importance of mitigating capsular contracture, a common complication that can lead to pain, deformity, and the need for revision surgery. For modified implants, core tests and coating stability assessments can ensure that the benefits of surface modification are not overshadowed by the long-term degradation. We conclude by encouraging more research on silicone implants and their biocompatibility, which will lead to better testing frameworks.

## Figures and Tables

**Table 1:** Summary of key historical events and regulatory shifts in the use and biocompatibility assessment of silicone implants (with a special focus on silicone breast implants). This timeline illustrates how biocompatibility controversies have propelled regulatory and methodological advancements, ensuring the safety of silicone implants.

Year/Period	Historical Event	Regulatory Action/Shift
1962	First silicone breast implant developed by Cronin and Gerow.	Food and Drug Administration (FDA) oversight limited; devices unregulated until 1976.
1965–1970s	Introduction of orthopedic (e.g., finger joints) and penile silicone implants.	1976 Medical Device Amendments classify implants as Class III *.
1980s	Rise in autoimmune concerns; reports of leakage and granulomas.	FDA begins adverse event monitoring; ISO standards drafted.
1992	FDA moratorium on silicone gel breast implants due to safety fears.	Moratorium restricts cosmetic use; mandates clinical trials.
1995	Dow Corning bankruptcy amid lawsuits over autoimmune claims.	Increased FDA scrutiny: epidemiological studies required.
2006	Studies debunk systemic risks; cohesive gels emerge (filled with a thick, semi-solid silicone gel that retains its form; more durable and less likely to leak).	FDA lifts moratorium; approves modern implants with labeling.
2010	Poly Implant Prothèse (PIP) scandal exposes fraudulent industrial silicone use.	European Union (EU) tightens certification; global recalls initiated.
2019	BIA-ALCL linked to mostly surface-textured implants.	FDA recalls specific devices; enhanced post-market surveillance.
2025	Advances in nanocomposite silicones (silicone matrix reinforced with nanoscale particles); ongoing breast implant illness (BII) debates.	Updated ISO 10993 and EU Medical Device Regulation (MDR) emphasize AI-integrated testing.

* Class III medical devices are the highest-risk devices, typically life-sustaining or life-supporting, and are implanted in the body, posing the highest potential for illness or injury. Due to this high risk, they require the most stringent regulatory oversight, such as pre-market approval from the FDA, to ensure their safety and effectiveness.

**Table 2:** Studies utilizing chemical methods to characterize the biocompatibility of silicone implants.

Aim of Study	Method or Test	In Vitro or In Vivo	Silicone Implant Type	Function Tested	References
Examine chemical structure of carbon ion-implanted silicone rubber	FTIR	In vitro	Silicone rubber	Chemical stability	[65]
Confirm presence of collagen on plasma-activated PDMS surfaces	FTIR	In vitro	PDMS	Surface modification	[66]
Characterize multilayer coatings on silicone implants	FTIR	In vitro	Silicone	Surface modification	[67]
Characterize novel silicone material modified with carboxybetaine ester analog	FTIR	In vitro	Silicone	Protein adsorption, Bacterial adhesion	[68]
Analyze chemical composition of silicone breast implant surfaces	Raman spectroscopy	In vitro	Silicone breast implant	Surface properties	[6]
Study surface chemistry of biomimetic silicone implants	Raman spectroscopy	In vitro	Silicone	Surface properties	[69]
Analyze surface chemistry of RTV silicone rubber after negative-ion implantation	Raman spectroscopy	In vitro	RTV silicone rubber	Surface modification	[70]
Confirm presence of hydrogel components on 3D-printed silicone implants	EDS	In vitro	3D-printed silicone	Surface modification	[71]
Detect silicone oligomers and lipids in explanted silicone breast implants	NMR	In vivo	Silicone breast implant	Degradation processes	[72]
Investigate dynamics of proteins adsorbed onto silicone surfaces	NMR	In vitro, In vivo	Silicone	Protein interactions	[73]
Identify LMW silicones in silicone gel of explanted PIP prostheses	GC-MS	In vivo	Silicone gel	Chemical stability	[58]
Detect volatile compounds and LMW silicones released from silicone implants	GC-MS	In vitro	Silicone	Chemical stability	[58]
To improve the cell compatibility of PDMS via chemical coating with tannic acid	XPS XPS elemental composition analysis		PDMS	Chemical group composition Elemental composition	[74,75]

**Table 3:** Comparison of various chemical methods used to analyze silicone implants biocompatibility. The scale used to standardize the level of sensitivity is provided in brackets in the second column. For example, the FTIR sensitivity is ~1% *w*/*w*, which is by definition less sensitive than NMR at 0.1% *w*/*w*.

Method	Sensitivity vs. Other Methods	Advantages	Limitations
FTIR	Moderate (~1% *w*/*w*); lower than NMR, MS methods	Rapid, accessible, identifies functional groups	Poor for traces; sample prep needed; matrix interference
NMR	High (~0.1% *w*/*w*); better than FTIR, Raman	Structural elucidation, high specificity	Expensive; not trace-sensitive; requires deuterated solvents
Raman	Moderate (~0.5% *w*/*w*); higher spatial than FTIR	Non-destructive, surface mapping	Fluorescence interference; lower sensitivity for some analytes
GC-MS	Very high (<1 ng/g); superior for volatiles	Precise quantification of LMWS leachables	Limited to volatiles; derivatization required; destructive
LC-MS	Very high (sub-ppm); best for non-volatiles	Broad coverage, high throughput	Costly; complex optimization; ionization artifacts
HPLC	Moderate (~ppm); lower than GC/LC-MS	Simple, cost-effective for screening	Low specificity without detection coupling; misses traces
ICP-MS	Ultra-high (<1 ng/L); best for trace elements	Quantitative elemental impurities detection	Destructive; matrix effects; no organic info
XPS	High (~0.1 atomic%); surface vs. bulk ICP-MS	Surface chemistry states, non-destructive	Shallow depth; vacuum required; expensive
EDS	Low (~0.5% *w*/*w*); less than ICP-MS, XPS	Elemental mapping with SEM; quick	Semi-quantitative; poor for light elements/traces
ToF-SIMS	High (~ppm surface); rivals MS for molecules	Molecular imaging, high resolution	Non-standard; costly; quantitative challenges

**Table 4:** Studies performed to characterize the biocompatibility of silicone implants using physical methods.

Aim of Study	Method or Test	In Vitro or In Vivo	Silicone Implant Type	Function Tested	Reference
Measure nanoscale roughness and correlate with cell adhesion on PDMS surfaces.	Atomic Force Microscopy (AFM)	In vitro	PDMS Implant	Nanoscale roughness and cell adhesion	[108]
Study nanoscale roughness and cell adhesion on nanostructured PDMS.	AFM	In vitro	PDMS Implant	Nanoscale roughness and cell proliferation	[109]
To improve the cell compatibility of PDMS via chemical coating with tannic acid	AFM Water contact angle measurement	In vitro	PDMS	Surface morphology Wettability	[75]
To investigate the interaction between fibroblasts and silicone breast implant surfaces	Confocal laser scanning microscope Water contact angle measurement	In vitro	Mentor siltex surface Mentor smooth surface Allergan Biocell textured surface Allergan smooth surface	Surface roughness Wettability	[110]
Analyze wettability and surface chemistry of PDMS implants.	Contact Angle Measurement	In vitro	PDMS Implant	Wettability and surface chemistry	[111]
Evaluate wettability and cell attachment on silicone implants with hydrophilic coatings.	Contact Angle Measurement	In vitro	Silicone Implant	Wettability and cell attachment	[112]
Assess fatigue resistance of silicone joint implants.	Dynamic Mechanical Testing	In vitro	Silicone Joint Implant	Fatigue resistance and stiffness	[113]
Monitor silicone breast implants for rupture and capsular contracture.	High-Resolution Ultrasound	In vivo	Silicone Breast Implant	Implant integrity and capsular contracture	[114]
Ultrasonographic Identification of Shell Surface Types in Commercially Available Silicone Gel–Filled Breast Implants	High-Resolution Ultrasound	In vivo	Silicone Implant	Tissue integration and inflammation	[115]
Monitor long-term integration of silicone implants in patients.	High-Resolution Ultrasound	In vivo	Silicone Implant	Capsular contracture and adverse effects	[116]
Characterize surface topography of silicone implants at micro and nano scales.	SEM and AFM	In vitro	Silicone Implant	Surface topography and biological responses	[117]
Examine surface morphology and detect biofilms on explanted silicone breast implants.	Scanning Electron Microscopy (SEM)	In vivo	Silicone Breast Implant	Surface degradation and biofilm detection	[118]
Characterize mechanical behavior of silicone materials for long-term stability.	Tensile Testing and Dynamic Mechanical Testing	In vitro	Silicone Implant	Mechanical stability and viscoelastic properties	[119]
Determine mechanical properties of PDMS formulations for facial implants.	Tensile Testing	In vitro	PDMS Implant	Mechanical strength and flexibility	[120]

**Table 5:** Summary and comparison of physical methods/techniques used for silicone implant biocompatibility characterization. The level or scale of sensitivity of the physical methods/techniques compared to each other is shown in brackets; for example, DMA sensitivity is very high at approximately 0.1 MPa, which is superior to tensile testing at approximately 1 MPa.

Method	Sensitivity vs. Other Methods	Advantages	Limitations
Tensile Strength Testing	High (~1 MPa); comparable to compression	Simple, standardizes durability metrics	Static; ignores viscoelasticity, cyclic loads
DMA	Very high (~0.1 MPa); superior to tensile	Dynamic frequency/temperature sweeps	Requires complex setups; sample geometry limits
Fatigue Testing	Moderate (~10^4^ cycles); better than static	Simulates in vivo stresses	Time-intensive; endpoint variability
Compression Modulus	High (~0.5 MPa); similar to tensile	Mimics palpability, clinical relevance	Limited to isotropic materials; no shear data
SEM	High (~1 nm lateral); lower vertical than AFM	High-depth imaging, qualitative topography	Vacuum/destructive prep; charging artifacts
AFM	Ultra-high (~0.1 nm vertical); best nanoscale	Force mapping, quantitative adhesion	Slow scanning; tip contamination
Contact Angle Goniometry	High (~1°); wettability-specific	Rapid surface energy assessment	Static; droplet evaporation errors
Optical Profilometry	Moderate (~0.1 μm lateral); faster than AFM	Non-contact 3D mapping	Limited resolution for nano-features
DSC	Very high (~0.1 °C); thermal events precise	Detects transitions, endothermic/exothermic	Small samples; ignores mass changes
TGA	High (~0.1% mass); complementary to DSC	Quantifies decomposition/volatiles	No phase info; inert atmosphere dependency
Accelerated Aging	Moderate (property shifts ~10%); holistic	Simulates long-term exposure quickly	Extrapolation uncertainties; accelerated rates
DMTA	High (~0.5 °C + 0.1 MPa); integrates thermal/mech	Comprehensive viscoelastic-thermal profile	Multifunctional equipment; data complexity

**Table 6:** Studies involving in vitro characterization methods of silicone implant biocompatibility.

Aim of Study	Method or In Vitro Test	Cell Type	Silicone Implant Type	Cell Function Tested	Reference
Investigate the effect of microgroove structures on PDMS-based silicone implants on biocompatibility	Cell Counting Kit-8, EdU, immunofluorescence, mass spectrometry	Fibroblasts	PDMS with microgrooves	Cell proliferation, cytoskeletal organization, protein adsorption	[160]
Evaluate the biocompatibility of modified surfaces of silicone breast implants (implant surfaces coated with polyacrylic acid and fibronectin)	Cytotoxicity assays, XTT assay, morphologic description	Murine L-929 fibroblasts	Silicone breast implant foils	Cytotoxicity, cell proliferation, cell morphology	[161]
Assess the cytotoxicity of chemically treated silicone materials (zinc diethydithiocarbamate (ZDEC) and benzalkonium chloride (BAK) were deposited on silicone surfaces at different concentrations)	Fluorescent viability dyes, confocal microscopy, resazurin assay	Human lens epithelial cells (HLEC)	Silicone surfaces	Cytotoxicity, cell viability	[162]
Evaluate the effect of collagen grafting on silicone via polydopamine on cell adhesion	Cell adhesion assay	Mesenchymal stem cells	Silicone	Cell adhesion	[46]
Assess the biocompatibility of collagen and polydopamine coated PDMS	Long-term culture, proliferation and morphology assays	Fibroblasts	PDMS	Cell proliferation, cell morphology	[163]
Investigate the influence of PDMS surface topography on fibroblast behavior	Cell adhesion assay, SEM, MTT assay	Human dermal fibroblasts	PDMS	Cell adhesion, cell proliferation	[69]
Examine the effect of nanotextured silicone surfaces on cell viability and morphology	Alamar Blue assay, SEM	Murine L-929 fibroblasts	Silicone breast implants (nanotextured)	Cytotoxicity, cell morphology	[164]
Examine the effect of various silicone implant surfaces on fibroblast morphology, proliferation, adhesion and capsular formation in rat models	CCK-8 Western blotting Immunofluorescence	Human fibroblasts	Silicone breast implants- -smooth surface; textured surface 1; Textured surface 2	Cell proliferation Cell adhesion Cell morphology Cytoskeletal organization	[165]
To improve the cell compatibility of PDMS via chemical coating with tannic acid	BCA assay MTT assay Calcein AM staining Annexin V apoptosis assay FITC-Phalloidin staining	Human liver cancer cells	PDMS	Protein adsorption Cell proliferation Cell adhesion Cytotoxicity Cell Morphology	[75]
To delineate the interplay between cells and silicone breast implant surfaces	Trypsin-mediated cell detachment	Human skin fibroblasts	Mentor siltex surface Mentor smooth surface Allergan Biocell textured surface Allergan smooth surface	Cell adhesion	[110]

**Table 8:** Animal Implantation studies used in assessment of silicone implant biocompatibility.

Aim of Study	Method or In Vivo Test	Animal Model Used	Silicone Implant Type	Function Tested	Reference (Title and Date)
Evaluate biocompatibility of silicone gel implants	Subcutaneous implantation	Rat	Silicone gel	Inflammation, encapsulation	[37]
Compare inflammatory response in male and female mice	Subcutaneous implantation	Mouse	PDMS	Inflammation	[211]
Evaluate biocompatibility of silicone breast implants	Implantation	Pig	Silicone breast implant	Inflammation, encapsulation	[11]
Evaluate long-term stability and immune response	Implantation	Rat	Silicone implant	Long-term stability, inflammation	[212]
Evaluate biocompatibility with immunomodulatory hydrogels	Implantation	Pig	Silicone implant	Inflammation	[71]
To assess the effect of silicone implant surfaces on fibroblast gene expression and capsular formation in rat models	Capsular formation-Hematoxylin and Eosin Masson trichome Immunohistochemical staining High resolution ultrasound	Rats		Collagen content Capsule formation	[165]

**Table 9:** Summary and comparison of biological methods/techniques used for silicone implant biocompatibility assessment. The Table shows the method/assay sensitivity versus other methods/techniques (in brackets) as well as the advantages and limitations.

Method/Assay	Sensitivity vs. Other Methods/Assay	Advantages	Limitations
MTT Assay	High (~10% viability); >LDH for metabolism	Rapid, quantitative, high-throughput	Indirect (metabolic bias); 2D-limited
LDH Release	High (~5% damage); >MTT for membrane	Direct cytotoxicity measure	Enzyme instability; no apoptosis distinction
Hemocompatibility	Moderate (~1000 platelets/mm^2^); <flow cytometry	Assesses thrombosis risk	Blood variability; static conditions
Ames/Comet Genotoxicity	High (~2-fold mutation); >histology for DNA	Screens mutagens early	Bacterial bias; misses epigenetic changes
Cytokine ELISA	Very high (~pg/mL); >RT-PCR for proteins	Quantifies inflammation	Snapshot only; costly multiplexing
Macrophage Polarization	High (~5% expression); >ELISA for cells	Models FBR initiation	Culture artifacts; donor variability
In vivo Implantation	Moderate (~10 μm capsule); >in vitro for systemic	Holistic chronic response	Ethical/costly; species differences
Histology (H&E)	High (~1 cell/HPF); >IHC for morphology	Spatial tissue insights	Qualitative; labor-intensive
Immunohistochemistry	Very high (~5% shifts); >histology for markers	Specific phenotype detection	Antibody variability; semi-quantitative
3D Skin Equivalent	High (~10 pg/mL cytokines); >2D for relevance	Mimics tissue architecture	No immune cells; short-term
Organ-on-a-Chip	High (~0.1 dyne/cm^2^ shear); >3D for dynamics	Simulates flow/interactions	Complex setup; non-standardized

## Data Availability

No new data were created or analyzed in this study.

## Abbreviations

3D: three-dimensional; AFM: atomic force microscopy; AI: Artificial Intelligence; ALCL: anaplastic large cell lymphoma; ANVISA: Agência Nacional de Vigilância Sanitária; ASPS: American Society of Plastic Surgeons; ATR-FTIR: Attenuated total reflectance-Fourier transform infrared spectroscopy; BII: breast implant illness; CCL2: chemokine C-C motif ligand 2; CD: cluster of differentiation; CFD: Computational fluid dynamics; CNN: Convolutional Neural Networks; CTGF: Connective tissue growth factor; DCF: Dichlorofluorescein; DSC: Differential scanning calorimetry; E: elastic modulus; ECM: extracellular matrix; EDS: Energy dispersive X-ray spectroscopy; ELISA: Enzyme-linked immunosorbent assay; EMA: European Medicines Agency; EU: European Union; ESI: Electrospray ionization; FBR: foreign body response; FDA: Food and Drug Administration; FEA: Finite element analysis; FTIR: Fourier transform infrared spectroscopy; FTT: Fast Fourier Transform; GC-MS: gas chromatography-mass spectrometry; GLP: Good Laboratory Practice; HA: Hyaluronic acid; HLEC: Human lens epithelial cells; HPLC-MS: High-performance liquid chromatography-Mass Spectrometry; HRUS: High-resolution ultrasound; IL: Interleukin; ISO: International Organization for Standardization; LDH: Lactate dehydrogenase; LVI: Large-volume injection; MALDI-TOF: Matrix-assisted laser desorption/ionization time of flight; MAS-NMR: magic-angle spinning-nuclear magnetic resonance; MDR: Medical Device Regulation; ML: Machine Learning; MRI: Magnetic Resonance Imaging; NIR: Near infra-red; NLP: Natural language processing; NMPA: National Medical Products Administration; NMR: nuclear magnetic resonance spectroscopy; PDMS: polydimethylsiloxane; PEG: poly (ethylene glycol); PE-UHMW: Ultra-high molecular weight polyethylene; PFT: Peak Force Tapping; PGA: poly-glycolic acid; PIP: Poly implant protheses; PLA: poly-lactic acid: PLGA: poly-lactic-co-glycolic acid; PLL: poly-l-lysine; PMAA: poly (methacrylic acid); RGD: arginine–glycine–asparagine; ROS: Reactive oxygen species; SEM: scanning electron microscopy; α-SMA: Alpha smooth muscle actin; TGF-β: Transforming growth factor β; TNF-α: Tumor necrosis factor α; ToF-SIMS: Time-of-Flight Secondary Ion Mass Spectrometry; TUV SUD: Technischer Überwachungsverein/Technical Inspection Association; UPLC: Ultra-performance liquid chromatography; VEGF: Vascular endothelial growth factor; WCA: Water contact angle; WHO: World Health Organization; XPS: X-ray photoelectron spectroscopy; XRD: X-ray diffraction.
